# Hydrolytic, Thermal, and Electrochemical Stability of Thiol- and Terminal Alkyne-Based Monolayers on Gold: A Comparative Study

**DOI:** 10.1021/acs.langmuir.4c05211

**Published:** 2025-03-01

**Authors:** Zhen Yang, Sidharam P. Pujari, Rachel Armstrong, Klaus Mathwig, Floris P. J. T. Rutjes, Maarten M. J. Smulders, Han Zuilhof

**Affiliations:** † imec within OnePlanet Research Center, Bronland 10, 6708 WH Wageningen, The Netherlands; ‡ Laboratory of Organic Chemistry, Wageningen University and Research, Stippeneng 4, 6708WE Wageningen, The Netherlands; § School of Pharmaceutical Sciences and Technology, Tianjin University, 92 Weijin Road, Tianjin 300072, P. R. China; ∥ College of Biological and Chemical Engineering, Jiaxing University, Jiaxing 314001, P. R. China; ⊥ Institute for Molecules and Materials, Radboud University, Heyendaalseweg 135, 6525 AJ Nijmegen, The Netherlands

## Abstract

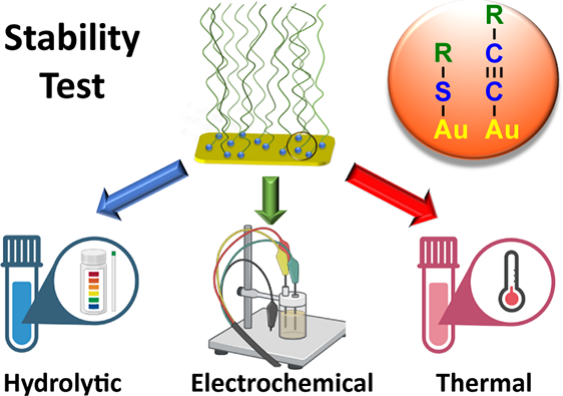

The terminal alkyne–Au interaction is emerging as a promising adsorbing bonding motif for organic monolayers, allowing it to be used for installing antifouling layers and/or recognition elements on gold surfaces for biosensing applications. In contrast to the well-known thiol-on-gold monolayers, the long-term hydrolytic, thermal, and electrochemical stability of the alkyne–Au bond remains relatively unexplored. Insight into these is, however, essential to deliver on the promise of the alkyne–Au bond for (bio)sensing applications, and to see under which conditions they might replace thiolate–gold bonds, if the latter are insufficiently stable due to, e.g., biological thiol exchange. Therefore, these stabilities were investigated for monolayers on Au substrates formed from 1-octadecanethiol and 1-octadecyne. Additionally, monodentate and tridentate alkyne-based adsorbates were designed to investigate the effect of multivalency on the stability. The hydrolytic stability over time in four aqueous media and the thermal stability in air were evaluated using static water contact angle measurements and X-ray photoelectron spectroscopy. Electrochemical oxidative desorption potentials were also assessed by cyclic voltammetry. All three tests indicate that the monovalent terminal alkyne monolayers on gold are slightly less stable than their thiolate analogs, which we could attribute to a lower packing density but still sufficiently stable to be applied in biosensing in the gut, while multivalency can further improve this. Our work provides insight into the stability of terminal alkynes under different conditions, better enabling the use of terminal alkyne–Au interactions in biosensors.

## 1 Introduction

Biosensors are gaining momentum in a wide range of applications, including clinical research, environmental monitoring, and medical diagnostics, as they often combine favorable properties such as real-time, selective, and sensitive analysis.^1^ The sensing components are the focus of biosensor development both in academic and industrial research. Despite the efforts dedicated to improving sensitivity and accuracy for detecting and analyzing target analytes, a persistent challenge is biofouling, which typically exists in complicated biological matrices such as blood, feces, extracellular fluids, and intestinal fluids, hindering further development and practical applications.^2−4^ Modifying the sensing part with an antifouling coating is widely applied as an effective strategy to prevent the nonspecific adsorption of biomolecules and microorganisms.^5−9^ Organic monolayers are frequently employed as interfaces between sensing substrates and the surrounding environment. They offer advantages such as controlled reactivity, easy preparation, and the possibility to tailor the surface properties.^10^ In electrochemical sensors, noble metals such as gold, silver, or platinum are widely used as electrode materials due to their excellent conductivity and chemical inertness.^11−13^ (R–SH) can spontaneously attach and self-assemble on these substrates to form ordered self-assembled monolayers (SAMs).^14^ Therefore, in addition to their role as standard models to investigate surface and interfacial chemistry, thiolate SAMs have also been extensively applied for electrochemical sensors.^15^

The stability of coatings is crucial for biosensors’ performance, and poor resistance to ambient disturbances lowers the precision and accuracy of measurements.^1^ Hence, extensive research has been conducted to explore the stability of thiolate SAMs under various conditions, including exposure to air,^16,17^ aqueous solutions,^16,18^ organic solvents,^16^ biological media,^18^ and different temperature regimes.^19^ Although the thiolate SAM stability is affected by various factors, the oxidation of −SH group is generally recognized as the main cause of the deterioration of thiolate SAMs.^17,20^ Moreover, applying thiolate SAMs as biosensor interfaces also faces more challenges. For example, undesirable interference in the sensing process may occur due to the potential exchange of thiol ligands by abundant biological thiols under physiological conditions.^21,22^ A widely adopted solution to enhance the monolayer stability involves utilizing multidentate adsorbates.^23^ These compounds possess the ability to form multiple bonds with the substrate, resulting in more strongly attached monolayers, reducing the risk of detachment (by exchange) or degradation. Diverse multidentate thiol-based adsorbates on Au substrates have been constructed and tested, and appropriately designed multidentate monolayers have been proven to have enhanced stability compared to their monosubstituted analogs.^23−25^

In addition to the strategy of increasing the number of binding sites, tailoring the surface attachment properties can also be achieved by changing the functional headgroup. Recently, various Au–C interactions have been developed including covalent Au–C σ bonds,^26,27^*N*-heterocyclic carbenes on gold^28−30^ and the terminal alkyne–Au motif.^31,32^ Among them, terminal alkynes have been increasingly studied because of the ease of preparation of these anchoring molecules, their stability in air (precluding the necessity to add reducing agents), and the mild reaction conditions required for surface attachment.^31,33^ The bonding process between terminal alkyne and gold, including the fate of the terminal hydrogen, has been reported previously,^34^ suggesting that the mechanism involves the heterolytic deprotonation of the terminal hydrogen, and formation of an upright configuration on Au, with an accompanying weakened C≡C bond. Moreover, the displacement experiments conducted by Landis and co-workers showed that an 1-ethynyl-4-fluorobenzene monolayer on Au exhibited a higher resistance to displacement by 1-octadecanethiol compared to a 4-fluorobenzene-1-thiolate monolayer, due to the enhanced stability of the Au–C bond.^35^ Additionally, alkyne monolayers typically exhibited a higher electrochemical transmittance compared to thiolate monolayers, which could potentially facilitate biosensor fabrication by improving signal transmission during sensing processes.^35,36^ Furthermore, the terminal alkyne–Au bonding motif has been employed in biosensors. Recently, Tian’s group achieved the real-time mapping and accurate analysis of Fe^2+^ in biological conditions by attaching ferrocenyl endoperoxide carboxylic acid as a detecting unit to an Au electrode via Au–C≡C bonds, which exhibited superior performance and was less affected by biological thiol (5 mM of glutathione) compared to Au–S and Au–Se analogs.^37^

**Scheme 1:**
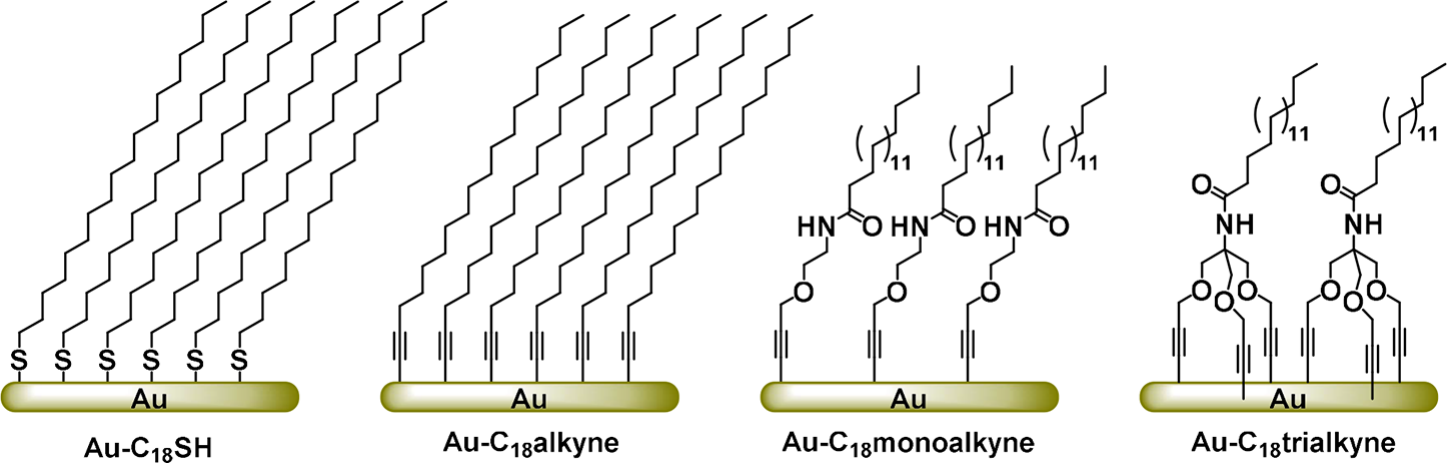
Illustration of the Organic Monolayers Formed by **C**_**18**_**SH**, **C**_**18**_**alkyne**, **C**_**18**_**monoalkyne**, or **C**_**18**_**trialkyne** on Gold **C**_**18**_**monoalkyne** and its trivalent counterpart **C**_**18**_**trialkyne** share similar chain lengths and functional groups; however, they differ in the number of binding sites.

Given this potential, detailed knowledge of the stability of Au–C≡C bonds under a variety of hydrolytic and thermal conditions is highly relevant, yet reports on this are still scarce. To close this gap, here we investigate the stability difference between the Au–C≡C and Au–S bonding motifs via a series of comparative experiments. First, we investigated and compared the hydrolytic stability over time of SAMs on Au made of 1-octadecanethiol (**C**_**18**_**SH**) or 1-octadecyne (**C**_**18**_**alkyne**, see Scheme 1) by XPS and SWCA. The media used included water, HCl (pH 3), and NaOH (pH 11), as well as simulated intestinal fluid (SIF) to investigate the stability of the monolayers in a complex biological ionic environment, providing a reference for their future application in the gut biosensors. Second, we assessed the thermal stability by analyzing monolayer samples after heating at an elevated temperature (80 °C). Finally, their electrochemical stability was probed via measured of their oxidative potentials by cyclic voltammetry (CV) measurements. In addition to investigating the effects of the bonding motif, the impact of the number of ligands of the terminal alkyne-based monolayers was also investigated. To this aim, a novel tridentate terminal alkyne adsorbate (**C**_**18**_**trialkyne**) was synthesized, along with its monodentate analog (**C**_**18**_**monoalkyne**, Scheme 1), and their stabilities were investigated.

## 2 Experimental Methods

### 2.1 Chemicals and Materials

Au substrates (200 nm thick Au layer on silicon) were obtained from Ssens (The Netherlands). 1-Dodecanethiol (**C**_**12**_**SH**) and 1-octadecanethiol (**C**_**18**_**SH**) were purchased from Sigma-Aldrich and directly used for surface modification without any purification. 1-Octadecyne (**C**_**18**_**alkyne**) was synthesized according to literature procedures.^38^ Di-*tert*-butyl decarbonate (Boc_2_O), hydrogen peroxide (H_2_O_2_, 30%), hydrochloric acid (HCl, 37%), sodium hydroxide (NaOH, pellets), and anhydrous triethylamine (TEA) were purchased from Sigma-Aldrich. Ethanolamine and sodium chloride (NaCl) were purchased from VWR Chemicals. Potassium hydroxide (KOH, pellets), magnesium chloride hexahydrate (MgCl_2_·6H_2_O) and potassium dihydrogen phosphate (KH_2_PO_4_) were purchased from Boom B.V. Propargyl bromide, trifluoroacetic acid (TFA) and stearoyl chloride were purchased from TCI. Potassium chloride (KCl), 2-amino-2-hydroxymethylpropane-1,3-diol (Tris), sulfuric acid (H_2_SO_4_, 96%), acetone, dimethylformamide (DMF), dichloromethane (DCM), ethanol, *n*-hexane, and methanol were purchased from Fisher Scientific. Petroleum ether (PE) and ethyl acetate (EA) were purchased from Labo-Scientific B.V. Ethanol used for surface modification was degassed by 3 consecutive freeze–pump–thaw (FPT) cycles. Anhydrous DCM was produced with a solvent purification system of Innovative Technology. Deionized water was produced with a Milli-Q Integral 3 system from Millipore, Molsheim, France. Simulated intestinal fluid without enzymes and Ca^2+^ (briefly named SIF in this paper) with pH of 7 was prepared according to literature procedures.^39^

Au disk electrodes (1.6 mm diameter), Pt wire (0.5 mm diameter) and Ag/AgCl (in 3 M KCl) were purchased from BASi. Diamond slurries (1 and 0.25 μm size), and alumina slurry (0.05 μm size) were purchased from Buehler (USA).

### 2.2 Synthesis of Adsorbates

*N*-(2-(prop-2-yn-1-yloxy)ethyl)stearamide (**C**_**18**_**monoalkyne**) and *N*-(1,3-bis(prop-2-yn-1-yloxy)-2-((prop-2-yn-1-yloxy)methyl)propan-2-yl)stearamide (**C**_**18**_**trialkyne**) were synthesized according to the protocol in the Supporting Information (Section S1).

### 2.3 Monolayer Preparation

Au flat surfaces were first sonicated separately in HPLC-grade *n*-hexane and acetone for 5 min in each solvent, and then rinsed with DCM and dried with Ar flow. Subsequently, the Au substrates were cleaned with piranha solution (H_2_SO_4_:H_2_O_2_ = 7:3 v/v) at room temperature (20 °C) for 10 min, then washed with copious amounts of Milli-Q (MQ) water, immersed in ethanol for 10 min, and dried using argon flow. [**Caution!***“Piranha” solution can react violently with organic substances, so please use with extreme caution.*] Au disk electrodes were first cleaned by sequential polishing with 1 and 0.25 μm diamond slurries, followed by 0.05 μm alumina slurry for a minimum of 1 min each. The electrodes were then rinsed with MQ water and ethanol, sonicated in MQ water for 3 min, immersed in piranha solution for 3 min, washed with copious amounts of MQ water, rinsed with EtOH, and dried with an Ar flow.

The Au substrates were immersed into 1 mL of ethanolic solutions of adsorbates (5 mM) in glass vials in an O_2_-free glovebox (SteDa Kunststoftechniek B.V.) with an O_2_ content of less than 1%. The thiolate monolayer formed at room temperature (20 °C) for 16 h, while the terminal alkyne monolayers were prepared at 60 °C for the same duration of time. Subsequently, the surfaces were rinsed with ethanol and *n*-hexane, then blown dried with an Ar flow. The monolayers formed on Au flat surfaces were characterized by SWCA and by XPS for quality control before hydrolytic and thermal stability tests.

### 2.4 Hydrolytic Stability Tests

The hydrolytic stability of the organic monolayers was evaluated through immersion in different media (1 mL media solution per surface), namely MQ-water, SIF (pH 7), HCl aqueous solution (pH 3), and NaOH aqueous solution (pH 11), for varying durations of 0, 1, or 7 days. All surfaces were placed with their Au side facing down on the vial bottom in the individual sealed glass vials to ensure complete contact with the solution. The vials were placed in the incubator shaker (Benchtop Innova 4080) at 25 rpm and a constant temperature of 25 °C. Before measurement, the surfaces were washed with MQ-water and then sonicated in ethanol for 10 s individually, and then the surfaces were subsequently rinsed with DCM and blown dry with an Ar flow. Three parallel surfaces were prepared for subsequent water contact angle and XPS measurements for each condition.

### 2.5 Thermal Stability Tests

To assess the thermal stability of the modified organic monolayers, the surfaces were placed in glass vials and exposed to various temperatures (80 °C) for 1 or 16 h in a heating oven (Carbolite Gero). The vials were not sealed and left open to the air. After heating, samples were washed with MQ-water and then sonicated in ethanol for 10 s, then rinsed with DCM and dried with an Ar flow. For each condition, three parallel surfaces were prepared for subsequent measurements of water contact angle and XPS.

### 2.6 Electrochemical Stability Tests

Electrochemical measurements were conducted on a PalmSens4 portable electrochemical workstation using an electrochemical cell from BASi in a three-electrode configuration. Ag/AgCl (in 3 M KCl) was used as the reference electrode and a Pt wire as the auxiliary electrode. The electrochemical oxidative potentials of the bare/modified Au disk electrodes (1.6 mm diameter) as working electrodes were measured by CV measurements in 0.1 M H_2_SO_4_ aqueous solution. The potential was scanned at 0.05 V/s starting from 0 to 1.5 V and then scanned back to 0 V.

### 2.7 Surface Analysis Tools

#### 2.7.1 SWCA Measurement

The Krüss DSA 100 contact angle goniometer was employed to measure the wettability of the samples. The volume of the deionized water drop is 2.0 μL. The tangent 2 fitting model was used for the contact angle measurement of all samples. Water contact angle measurements were performed before the XPS to ensure the quality of the monolayers.

#### 2.7.2 X-ray Photoelectron Spectroscopy

A JPS-9200 photoelectron spectrometer (JEOL, Japan) was used to perform all XPS measurements, with a monochromatic Al Kα X-ray source (10 kV and 20 mA). The analyzer pass energy was set to 50 eV for wide scans and 10 eV for narrow scans of C 1s. The incident X-ray angle was 10°, and the takeoff angle (φ) between the sample and detector was 80°. The measurements were processed at 1 × 10^–6^ Torr. All data were analyzed with CasaXPS software (version 2.3.26).

#### 2.7.3 Atomic Force Microscopy

Under ambient conditions, AFM imaging was performed using an MFP-3D-BIO AFM (Asylum Research, Oxford Instruments) operating in tapping mode. The imaging utilized aluminum reflex-coated NCHR-16 cantilevers (NANOSENSORS, Neuchatel, Switzerland) with a tip radius of <10 nm, a frequency of 330 kHz, and a force constant of 42 N/m. Images were captured at a resolution of 512 × 512 pixels with a scanning rate of approximately 1 Hz.

#### 2.7.4 Ellipsometry

The ellipsometric thickness of the monolayers was measured using an AccurionNanofilm_EP4 Imaging Ellipsometer (Park Systems), operating at 562.9–642.3 nm at an angle of incidence of 50° in the air at room temperature. Each sample was measured at three different areas, and the average values were reported. The acquired data were fitted using EP4 software employing a multilayer isotropic model that included the ambient, monolayer, and substrate, with a clean gold surface used as the substrate. The monolayers were characterized using a Cauchy model with a parameter of refractive index (*A* = 1.45).

## 3 Results and Discussion

### 3.1 Monolayer Preparations

#### 3.1.1 Au Substrate Cleaning

Choosing a proper surface cleaning method is crucial for the formation of high-quality monolayers since the Au substrate is easily contaminated. Therefore, the Au substrate cleaning approaches were first investigated. In brief, we explored two commonly used methods: Au substrates were cleaned either by immersing in piranha solution for 10 min,^40^ or by air plasma,^41^ followed by thorough washing with water and immersion in EtOH for 10 min. Both methods proved effective in reducing organic contaminants, as evidenced by the minor carbon content on the Au substrates (SI Figure S9). However, in contrast to the Au surface subjected to piranha solution, the Au surface treated with air plasma exhibited a higher oxygen content, which might be attributed to the presence of a partial oxide (Au_2_O_3_) thin layer on the Au substrate.^42−44^ Subsequently, 1-dodecanethiol (**C**_**12**_**SH**) was self-assembled to form a thiolate SAM (**Au–C**_**12**_**SH**) on both types of Au substrates inside an oxygen-free glovebox. The XPS spectrum revealed that the abundant oxygen content remained in the **Au–C**_**12**_**SH** formed on air plasma-cleaned Au substrate, while the oxygen content was greatly reduced for the Au surface cleaned with piranha solution. Therefore, we chose 10 min of piranha solution cleaning to eliminate the potential influence of oxygen on the monolayers’ stability.

#### 3.1.2 Monolayer Formation on Au Substrates

**Figure 1:**
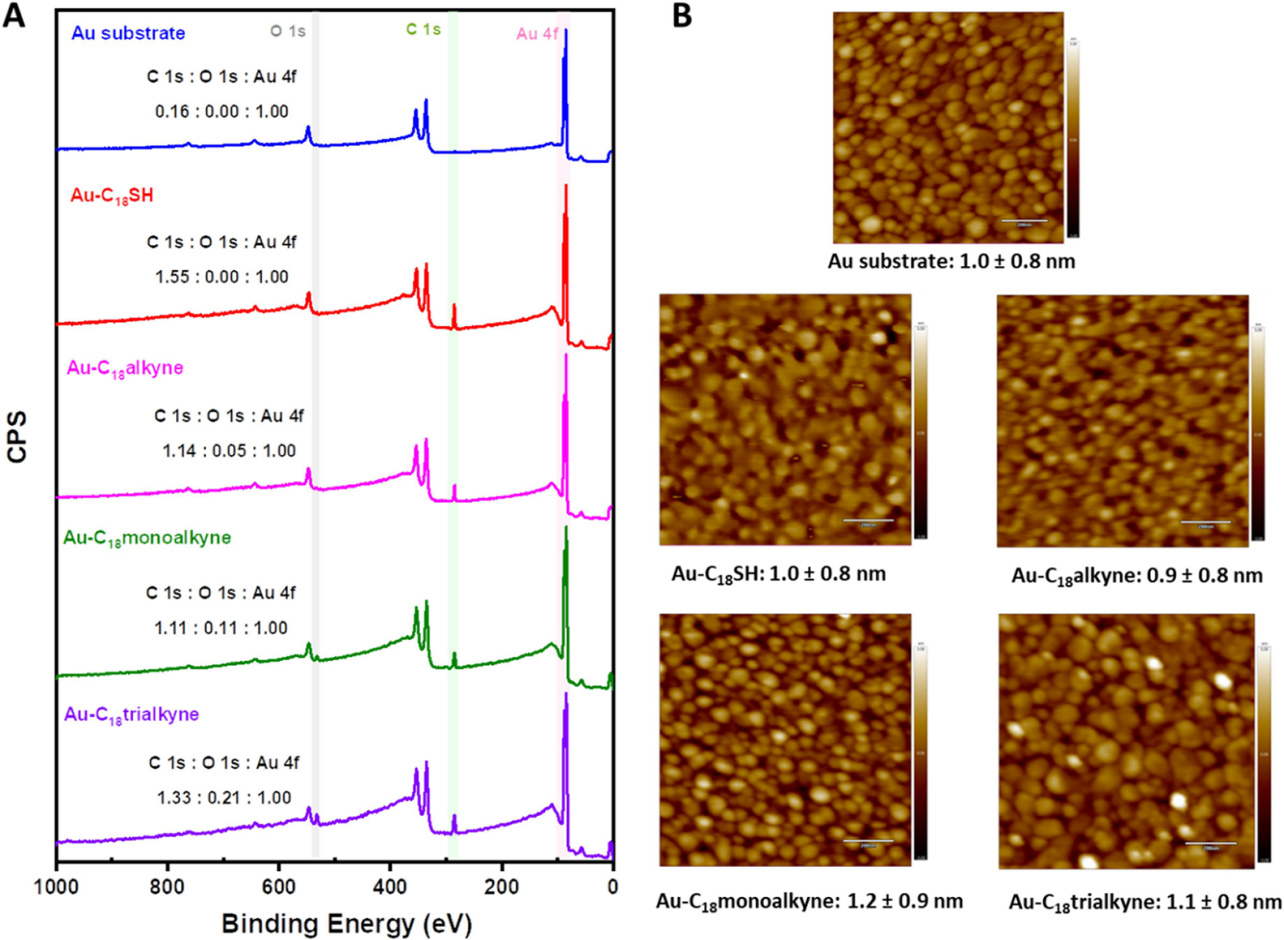
(A) XPS wide scan and (B) AFM tapping mode images (scale bar = 200 nm) of the piranha solution-cleaned Au substrate and four different monolayers formed on the Au substrate.

To compare the stability of thiolate and terminal alkyne self-assembled monolayers, 1-octadecanethiol (**C**_**18**_**SH**) and 1-octadecyne (**C**_**18**_**alkyne**) were first allowed to self-assemble on gold surfaces. Additionally, monodentate (**C**_**18**_**monoalkyne**) and tridentate (**C**_**18**_**trialkyne**) alkyne-terminated adsorbates were synthesized (SI Scheme S1), and analogously allowed to form monolayers on gold surfaces, to understand the impact of multidentate structures on the stability of terminal alkyne monolayers. Monolayers were prepared by immersing freshly cleaned Au substrates in 5 mM of ethanolic solutions of each of the four adsorbents for 16 h (Scheme 1). All surface modifications were carried out inside an oxygen-free glovebox to minimize oxidation of either the alkyne or thiol groups during the immobilization reaction; thiols were self-assembled at room temperature, and alkynes at 60 °C according to literature.^32^ The monolayers were first characterized by SWCA measurements, and subsequently analyzed with XPS to check the quality of the prepared monolayers (Table 1 and Figure 1A).

**Table 1:** Overview of Results of XPS and SWCA Characterization of Monolayers on Au Substrates

substrate-monolayer	adsorbate’s molecular formula	C 1s/Au 4f signal ratio^a^	SWCA^b^ (°)
**Au–C**_**18**_**SH**	C_18_H_38_S	1.55 ± 0.07	111 ± 1
**Au–C**_**18**_**alkyne**	C_18_H_34_	1.14 ± 0.09	102 ± 2
**Au–C**_**18**_**monoalkyne**	C_23_H_43_NO_2_	1.11 ± 0.03	101 ± 3
**Au–C**_**18**_**trialkyne**	C_31_H_51_NO_4_	1.33 ± 0.08	99 ± 2

a Averaged values of (at least) 3 samples are reported.

b Averaged values of (at least) 10 samples are reported.

The SWCA of **Au–C**_**18**_**SH** was 111 ± 1°, which was higher than the SWCA of **Au–C**_**18**_**alkyne**, determined at 102 ± 2°. The difference in wettability is attributed to a higher packing density in the **Au–C**_**18**_**SH** as compared to its **Au–C**_**18**_**alkyne** counterpart.^45^ This is borne out by a quantitative XPS analysis, in which the **Au–C**_**18**_**SH** revealed a higher C 1s/Au 4f signal ratio of 1.55 compared to the value for **Au–C**_**18**_**alkyne** at 1.14. Based on these two values, the relative packing density of the alkyne monolayer is 74% relative to its thiol counterpart. This difference in packing densities also shows up in the ellipsometric thicknesses: for **Au–C**_**18**_**SH** this is 2.2 ± 0.1 nm, which aligns with the reported data (2.3 nm)^25^ and its theoretical thickness (2.3 nm, Chem3D). For **Au–C**_**18**_**alkyne**, it is 1.8 ± 0.1 nm, which is slightly lower than its theoretical thickness (2.1 nm, Chem3D). Moreover, an O 1s/C 1s signal ratio (0.04 ± 0.03) was observed in **Au–C**_**18**_**alkyne**, consistent with the reported O 1s/C 1s signal ratio (<0.06) of terminal *n*-alkynes (HC≡C(CH_2_)_*n*_CH_3_, *n* = 5, 7, 9, and 11) SAMs on Au.^32^ This presence of oxygen can be explained by partial oxidation of the alkyne head groups even upon construction in a glovebox, contributing to reduced ordering and packing density, as previously reported.^32^ These observations are similar to findings in the literature, which reported that the packing density of an aromatic alkyne monolayer is ca. 63% of that of the corresponding aromatic thiol monolayers.^35^ Additionally, both **Au–C**_**18**_**monoalkyne** and **Au–C**_**18**_**trialkyne** displayed slightly lower SWCAs than **Au–C_18_alkyne**, in line with the presence of the polar amide group.

Next, the morphologies of formed monolayers were characterized by AFM (Figure 1B). The roughness of the four different monolayers did not exhibit significant changes compared to the freshly cleaned Au substrate (1.0 nm ± 0.8 nm), which indicates that the formed monolayers uniformly cover the gold substrate surface with excellent homogeneity.

### 3.2 Bonding Motif Comparison

#### 3.2.1 Hydrolytic Stability

As sensors (with their sensing surfaces) often operate while immersed in aqueous solutions, the hydrolytic stability of interfaces plays a crucial role in withstanding the challenges presented by complex matrices in which they should operate. To compare the hydrolytic stability of alkyl thiolate and terminal alkyne monolayers, modified Au surfaces were immersed in four different aqueous environments for a continuous duration of 7 days with constant agitation. These solutions were MQ-water, simulated intestinal fluid (SIF, pH 7) given our interest in developing gut biosensors, aqueous HCl solution (pH 3), and aqueous NaOH solution (pH 11). After 0, 1, and 7 days, the surfaces were taken out and were rinsed and dried, and changes in surface wettability were assessed through SWCA measurements, while XPS measurements were used to analyze the variation in the C 1s/Au 4f ratio to independently monitor changes in monolayer quality.^46,47^

**Figure 2:**
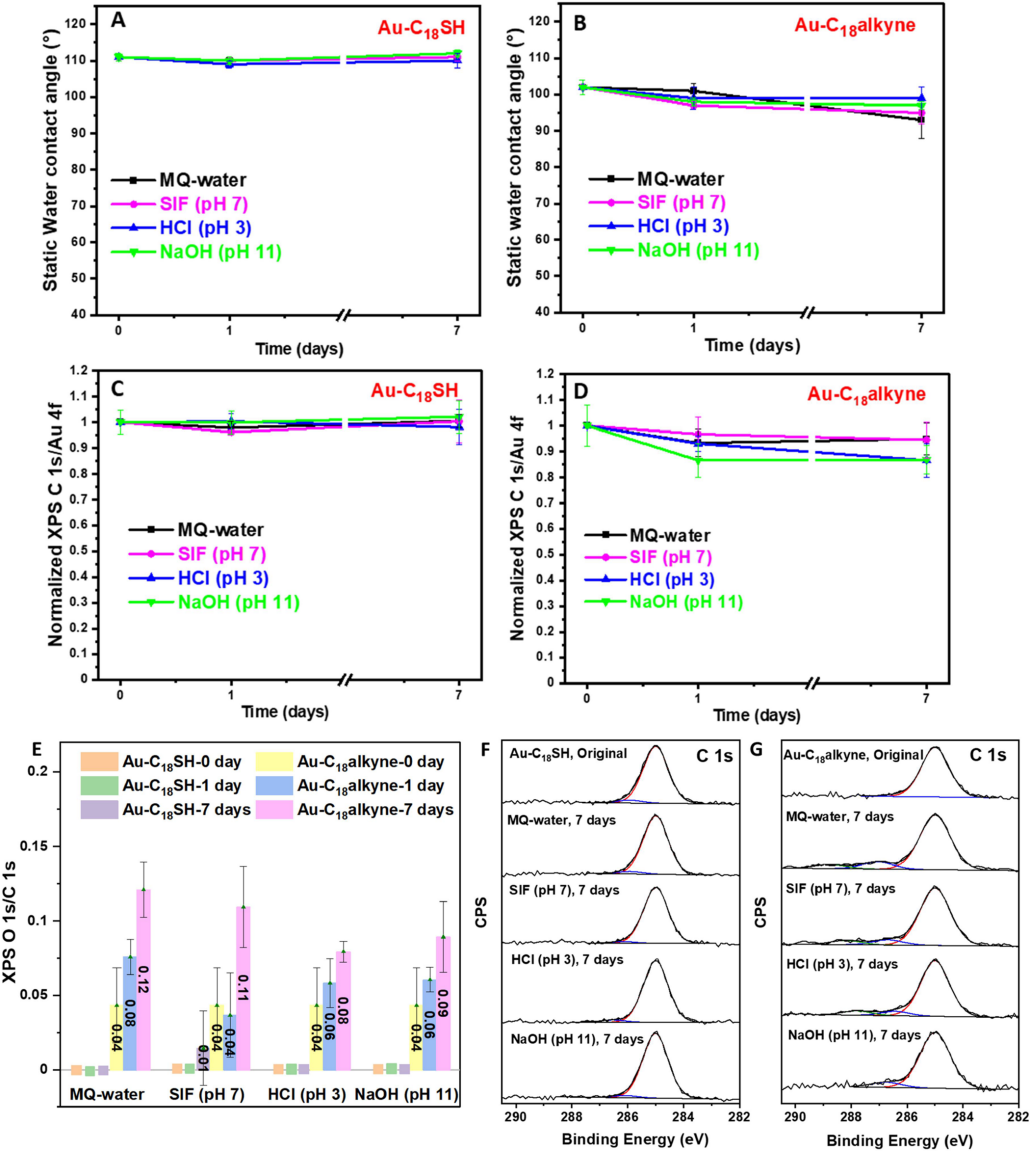
Hydrolytic stability of alkyl SAMs in various aqueous media. (A, B) SWCA measurements, (C, D) normalized XPS C 1s/Au 4f signal ratios, (E) XPS O 1s/C 1s signal ratios, and (F, G) C 1s XPS narrow-scan spectrum for **Au–C**_**18**_**SH** and **Au–C**_**18**_**alkyne**, respectively. Measurements were performed after taking out the samples at the indicated time points from MQ-water, SIF (pH 7), HCl solution (pH 3), and NaOH solution (pH 11).

The hydrolytic stability results of **Au–C**_**18**_**SH** and **Au–C**_**18**_**alkyne** as characterized by SWCA measurement are shown in Figure 2A,B. It was found that no significant changes in the surface wettability were observed for the **Au–C**_**18**_**SH** surfaces after immersion in the four different aqueous media for 7 days. This result is consistent with the reported good hydrolytic stability of **Au–C**_**18**_**SH**, which exhibited only 8 and 3° decreases in SWCA after immersion in 1 N HCl and 1 N NaOH for 1 month, respectively.^47^ For **Au–C**_**18**_**alkyne**, a slight decline in hydrophobicity could be observed after 7 days of immersion: the SWCA decreased to 93 ± 5° in MQ-water, to 95 ± 3° in SIF, to 99 ± 3° in HCl, and to 97 ± 1° in NaOH, respectively, from its original value of 102 ± 2°. The results indicated that some deterioration in the integrity of monolayers had occurred, but also that such changes are likely acceptable given the usage time of most biosensors, such as the transportation times of pill-embedded ingestible sensors traveling through the human gastrointestinal (GI) tract. The changes in the XPS C 1s/Au 4f signal ratio shown in Figure 2C,D for **Au–C**_**18**_**SH** and **Au–C**_**18**_**alkyne**, respectively, allow for a further understanding of the effect of immersion on the monolayer. Aligning with the stability results from the SWCA measurement, the C 1s/Au 4f signal ratios of **Au–C**_**18**_**SH** also remained constant over time, suggesting the monolayer was highly stable under all the investigated conditions. This corresponds to the reported research findings of other thiolates SAM on Au in Tris-buffered saline, which showed near-constant C 1s/Au 4f signal ratios over 7 days.^48^ For the **Au–C**_**18**_**alkyne** in all media a slight decrease in the C 1s/Au 4f signal ratio was observed from day 0 to day 7, with a good retention of the monolayer quality on both MQ water (95 ± 6% of original C 1s/Au 4f signal ratio) and SIF (94 ± 7%), whereas a week in HCl or NaOH decreased this ratio to 87 ± 7 and 87 ± 5%, respectively. This indicates two things: (1) the terminal alkyne SAMs are slightly less resistant to harsh conditions, such as acidic or basic solutions; (2) their stability is likely more than enough for pill-embedded ingestible sensors.

This difference in monolayer stability is attributed to the oxidation of the SAMs. Oxygen-containing species have been reported to form during the formation of acetylenylbenzene monolayers on gold substrates, possibly resulting from gold-catalyzed reactions with ambient oxygen.^49^ Although this process was suppressed by preparing terminal alkyne monolayers in an O_2_-free glovebox, achieving a low O 1s/C 1s signal ratio (0.04 ± 0.03), oxidation can still happen after SAM formation.^32^ Hence, we investigated the variation of O 1s/C 1s signal ratios in the two types of SAMs in the four different media over time. Figure 2E shows that the oxygen content in **Au–C**_**18**_**SH** remained almost unchanged over 7 days, likely due to the ordered structure of the **Au–C**_**18**_**SH** monolayer film enhancing its oxidation resistance.^17^ However, in the case of **Au–C**_**18**_**alkyne**, there were visible increases in the O 1s/C 1s signal ratios, which rose from their initial value of 0.04 ± 0.03 to values ranging from 0.08 ± 0.01 to 0.12 ± 0.02 in different media after 7 days. Apart from analyzing the O 1s/C 1s ratio in the survey scans, the corresponding C 1s narrow scans were also measured to better understand the (change in) monolayer composition. Figure 2F,G exhibits a comparison in the XPS C 1s narrow scan from these two monolayers. The C 1s narrow scan for **Au–C**_**18**_**SH** remained unchanged across four different aqueous media over 7 days, while for **Au–C**_**18**_**alkyne**, new carbon peaks with higher binding energies ranging between 285.5 and 289.0 eV appeared, which are assigned to oxidized carbon atoms.^49^ This comparison implies that terminal alkyne monolayers on Au exhibit a higher susceptibility to oxidation than thiolate monolayers, probably due to a lower oxidation potential of the Au–C≡C moiety (see below) and the lower packing density; the first is related to the nature of the weakened C≡C bonds caused by the back-donation of electrons from the d-orbitals of Au to the π* orbital of alkyne.^34,50^ In addition, the lower packing density of the formed monolayers may raise the likelihood of contact between oxidants (like O_2_) and the headgroup-substrate interface.^51^

#### 3.2.2 Thermal Stability

**Figure 3:**
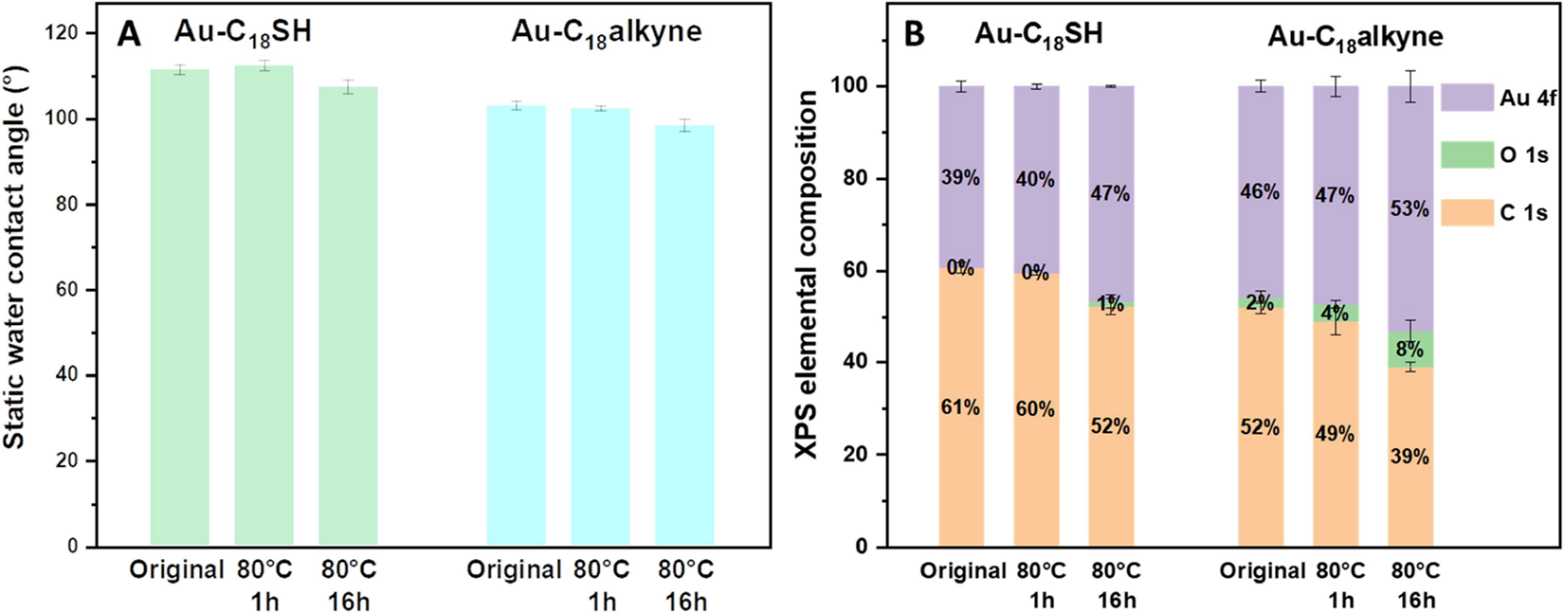
Thermal stability of alkyl SAMs at 80 °C. (A) SWCA and (B) XPS elemental composition for **Au–C**_**18**_**SH** and **Au–C**_**18**_**alkyne** before and after heating at 80 °C for 1 and 16 h, respectively.

Following the hydrolytical stability tests, the thermal stability of the monolayers was investigated under atmospheric conditions. Considering that most biosensors generally operate at relatively low temperatures (20–37 °C), SAMs were heated to 80 °C for 1 and 16 h, respectively, to rapidly obtain thermal stability data. After heating, the samples were cleaned by rinsing with MQ-water, sonicating in ethanol for 10 s, and then rinsing with DCM before being dried with Ar. Figure 3A shows that both **Au–C**_**18**_**SH** and **Au–C**_**18**_**alkyne** exhibited nearly unchanged SWCAs after 1 h at 80 °C. Analogously, Figure 3B indicates that the elemental compositions of **Au–C**_**18**_**SH** also remained constant. Meanwhile, for **Au–C**_**18**_**alkyne**, the XPS C 1s signal ratio exhibited slight changes after heating at 80 °C for 1 h, while the XPS O 1s signal increased from 2 ± 1 to 4 ± 1%, indicating that the SAM underwent slight oxidation at elevated temperatures. That all layers eventually oxidized became more obvious after heating for 16 h at 80 °C, when an obvious deterioration in both **Au–C**_**18**_**SH** and **Au–C**_**18**_**alkyne** was observed (see Figure 3B). This is further supported by the appearance of peaks at around 289.1 ev of oxygen-containing species in the C 1s narrow scan spectrum (see SI Figure S15). These data show that long-term storage at room temperature is preferred for both types of SAMs and that prolonged heating deteriorates the quality.

#### 3.2.3 Electrochemical Stability

**Figure 4:**
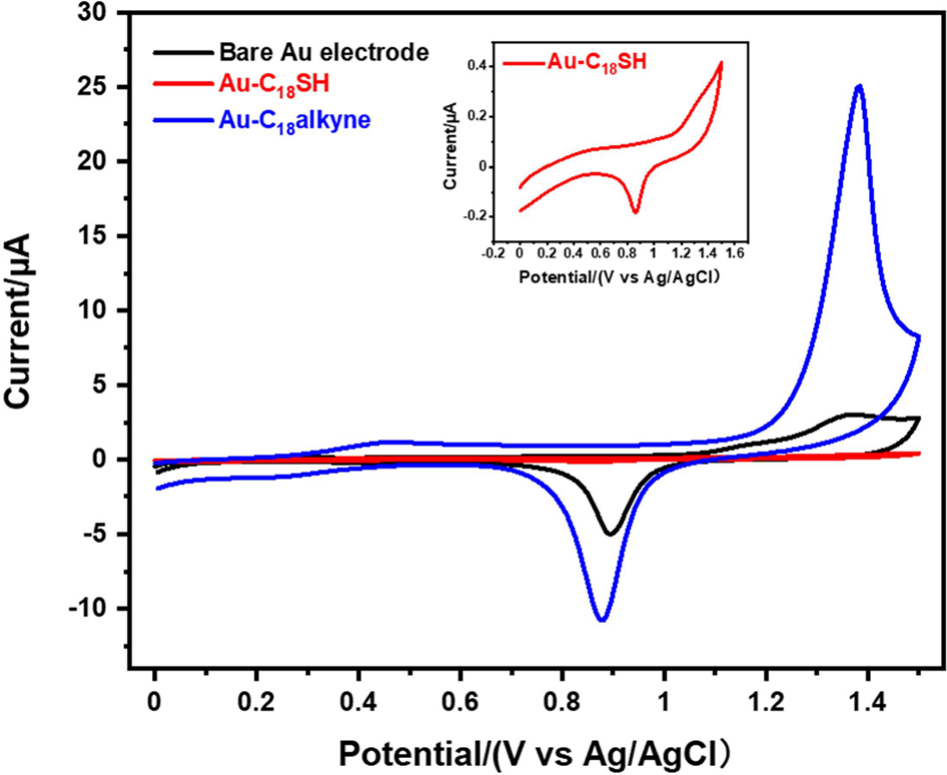
Cyclic voltammograms for **Au–C**_**18**_**SH** and **Au–C**_**18**_**alkyne** in an aqueous electrolyte of 0.1 M H_2_SO_4_ at a scan rate of 0.05 V/s.

SAM-modified Au electrodes were used as working electrodes in CV experiments to characterize their electrochemical stability, by sweeping from 0 to 1.5 V in 0.1 M aqueous H_2_SO_4_ solution to investigate and compare their oxidative desorption potentials. As shown in Figure 4, an oxidation peak was observed at ∼1.37 V for the bare Au electrode, corresponding to the oxidation of the Au substrate, while the reductive peak at ∼0.90 V during the reverse sweep is attributed to the electrochemical reduction of the oxidized Au substrate.^52^ CV scans of **C**_**18**_**SH**-modified electrodes show a lower current compared to bare Au electrodes, as the SAMs inhibit redox reactions. The significant oxidative current for **Au–C**_**18**_**SH** occurred at a higher potential compared to the oxidation peak of the Au substrate (>1.5 eV), consistent with the reported data for SAMs of 1-propanethiol.^52^**Au–C**_**18**_**alkyne** remained electrochemically inert up to ∼1.20 V, with the oxidation current peaking at around 1.38 V, in alignment with the electrochemical behavior of the reported conjugated alkyne-anchored monolayers.^36,53^ The oxidative peaks in **C**_**18**_**SH** and **C**_**18**_**alkyne** can most likely be attributed to the electrochemical oxidation of the monolayers themselves, together with the Au substrate oxidation process, as evidenced by significant reductive peaks of gold oxide during the reverse sweeping.^52−54^ Existing research suggests that thiols are mostly likely irreversibly oxidized to oxidized sulfur species (like R-SO^2–^),^55,56^ However, for terminal alkynes, there has been no detailed study on the oxidized species and the mechanism of oxidative desorption, and further investigation is required. In summary, the obtained oxidation potentials suggest that both thiolate and alkyne SAMs-modified interfaces remain stable in a wide anodic potential window, which benefits their application in electrochemical sensors.

In summary, (monovalent) thiolate and alkyne SAMs exhibit comparable hydrolytic and thermal stabilities, demonstrating the potential of alkyne-based SAMs on gold in biosensor applications. In addition, it has been reported in the literature, that alkyne-based monolayers are more resistant to displacement by organic thiols compared to their thiol analogs,^35,37^ further demonstrating their stability and derived possibility in complex media involving thiol-containing biomolecules (like proteins). Moreover, recent studies have demonstrated that alkyne monolayers on Au exhibit superior charge transfer compared to thiolate monolayers possibly due to their moderate packing density,^35,36^ or the extension of the conductive moiety by the C≡C group.^36^ This characteristic enhances their potential for application in sensor technology, particularly where efficient electron transfer is crucial, such as electrochemical sensors.

### 3.3 Bonding Number Comparison

To further increase the robustness of terminal alkyne-based interfaces by multivalency, we synthesized two adsorbates (see Scheme 1) with similar core and chain lengths, but varying numbers of alkyne binding sites. The straightforward synthesis primarily involved the amide formation between the appropriate amine and stearoyl chloride (see SI Scheme S1). These mono- and trivalent alkynes were used to make the corresponding monolayers under the same optimized formation conditions previously found for **Au–C**_**18**_**alkyne** (see above).

#### 3.3.1 Hydrolytic Stability

**Figure 5:**
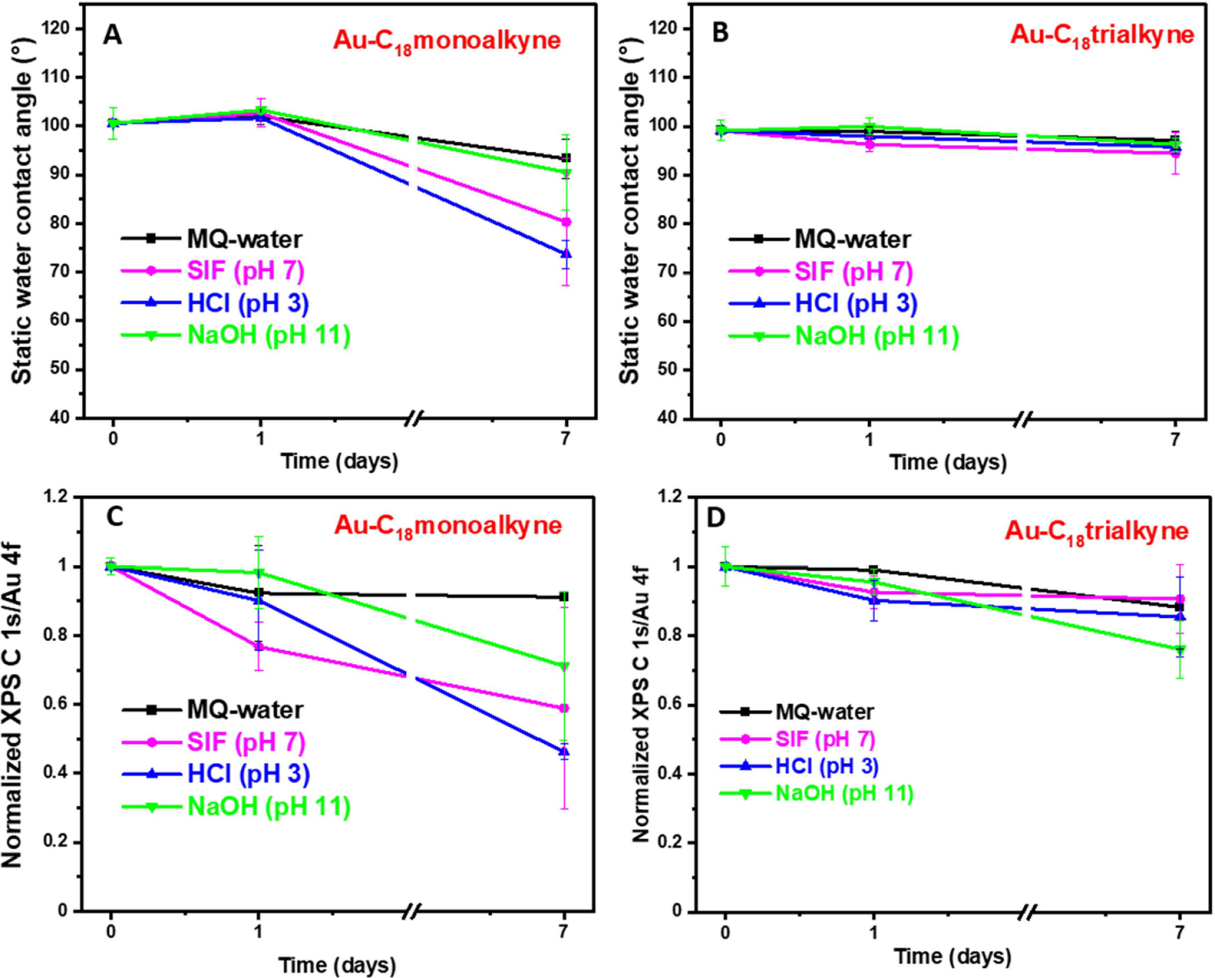
Hydrolytic stability of mono- and trivalent alkyne monolayers in various aqueous media. (A, B) SWCA and (C, D) normalized XPS C 1s/Au 4f signal ratios for **Au–C**_**18**_**monoalkyne** and **Au–C**_**18**_**trialkyne**, respectively. Measurements were performed after taking out the samples at the indicated time points from MQ-water, SIF (pH 7), HCl solution (pH 3), and NaOH solution (pH 11).

Hydrolytic stability tests were similarly conducted on these two monolayers, by immersion in MQ-water, SIF (pH 7), HCl solution (pH 3) or NaOH solution (pH 11) for up to 7 days. As shown in Figure 5B, the contact angles of **Au–C**_**18**_**trialkyne** remained practically constant over 7 days across four different solutions. In contrast, the SWCA of the **Au–C**_**18**_**monoalkyne** (see Figure 5A) showed a visible decrease from 1 to 7 days in all four different solutions. Specifically, the SWCA decreased from 101 ± 3 to 93 ± 4° in water, to 80 ± 13° in SIF, to 74 ± 3° in HCl, and to 91 ± 8° in NaOH. Additionally, XPS analysis was used to quantitatively monitor monolayer changes. The **Au–C**_**18**_**trialkyne** (see Figure 5D) showed relatively minor variation in the normalized XPS C 1s/Au 4f signal ratio (referenced to freshly prepared monolayers), remaining 88 ± 12% in MQ-water, 91 ± 10% in SIF, 85 ± 11% in HCl, and 76 ± 8% in NaOH after 7-day immersion. In contrast, the **Au–C**_**18**_**monoalkyne** (see Figure 5C) only displayed a stable ratio in MQ-water after 7-day immersion, with about 91 ± 1% of its original signal ratio maintained, but the monolayer was basically destroyed in the other media C 1s/Au 4f signal ratios between 29 and 54%. These results indicate that the trivalent binding drastically improved the hydrolytic stability of the alkyne-based monolayers in these different media, bringing it close to even the thiol-based monolayers.

#### 3.3.2 Thermal Stability

**Figure 6:**
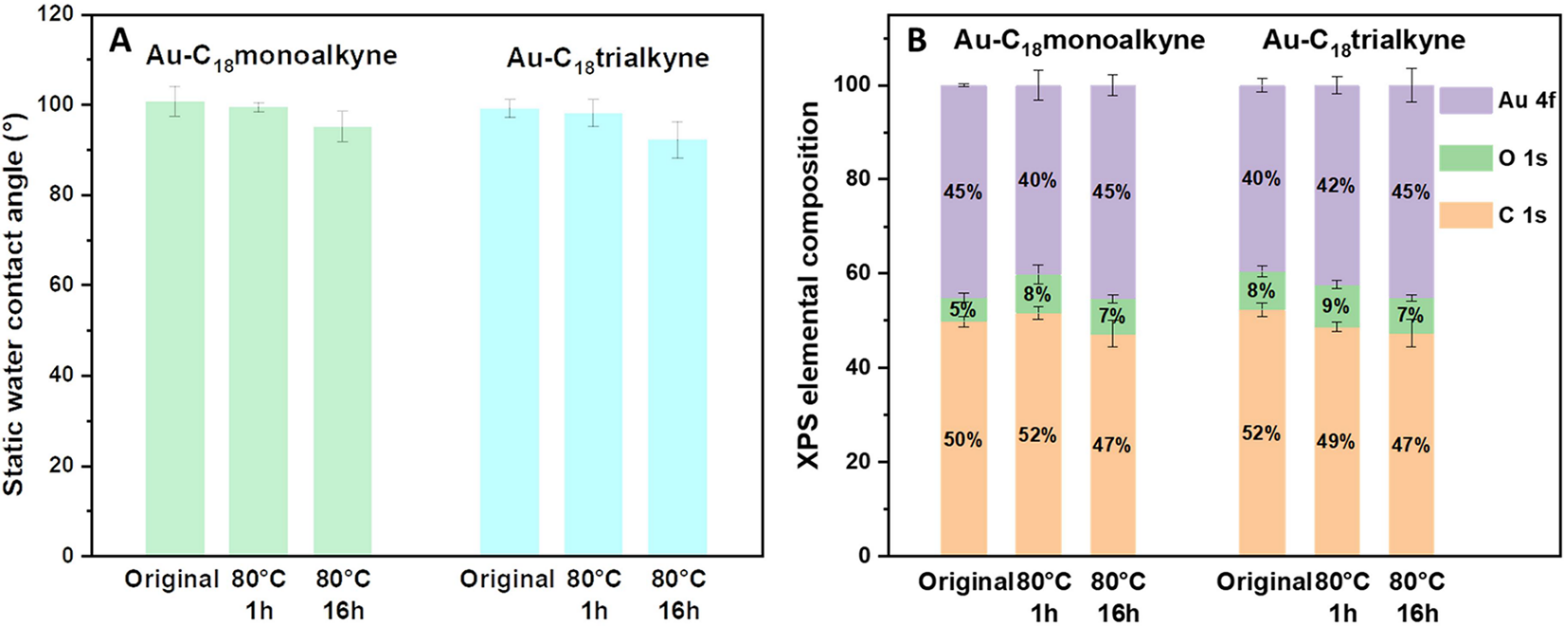
Thermal stability of mono- and trivalent alkyne monolayers at 80 °C. (A) SWCA measurements and (B) XPS elemental composition for **Au–C**_**18**_**monoalkyne** and **Au–C**_**18**_**trialkyne** heated at 80 °C for 1 and 16 h.

Figure 6A illustrates that both **Au–C**_**18**_**monoalkyne** and **Au–C**_**18**_**trialkyne** did not undergo large changes in wettability after being heated at 80 °C for 1 h and only a slight decrease after 16 h of heating. The same conclusion is also obtained more straightforwardly from the XPS elemental composition changes. The C 1s XPS spectrum (SI Figures S18 and S19) provided additional details: the peak area of peaks at ∼286.5 and ∼288.5 eV showed a slight increase compared to their original monolayers, indicating partial oxidation under heating conditions, likely forming more oxygen-containing species with C–O or C=O bonds. Interestingly, compared to the previously discussed **Au–C**_**18**_**alkyne**, not only the tridentate **Au–C**_**18**_**trialkyne** showed better heat resistance due to its multivalent binding, but the monodentate **Au–C**_**18**_**monoalkyne** also exhibited improved thermal stability. This may be attributed to the stabilizing effect of intermolecular hydrogen bonds formed within the **Au–C**_**18**_**monoalkyne** backbone due to the presence of amide bonds.^57,58^

#### 3.3.3 Electrochemical Stability

**Figure 7:**
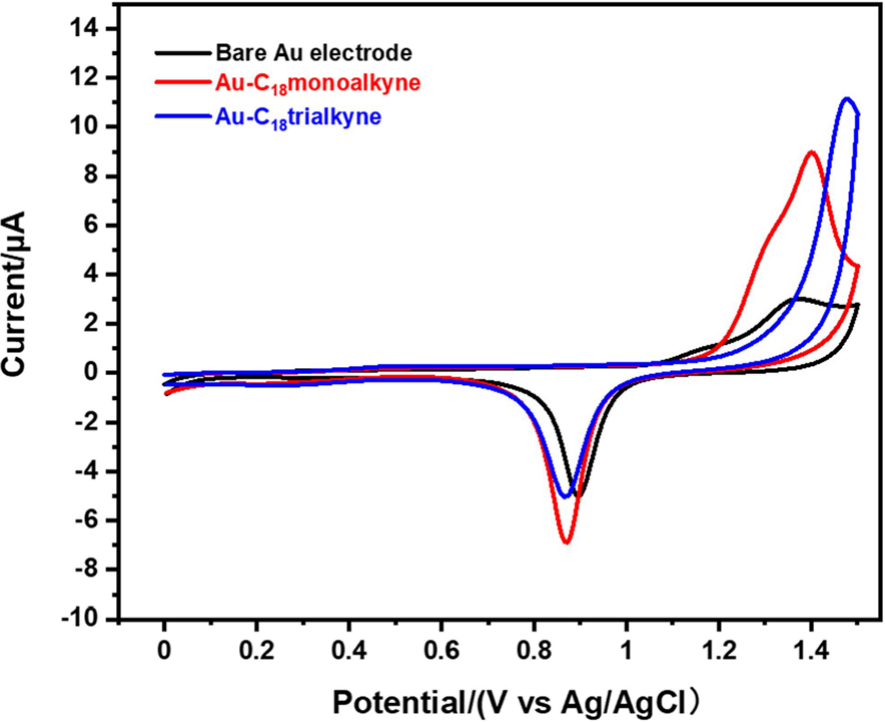
Cyclic voltammograms for **Au–C**_**18**_**monoalkyne** and **Au–C**_**18**_**trialkyne** in an aqueous electrolyte of 0.1 M H_2_SO_4_ at a scan rate of 0.05 V/s.

Cyclic voltammetry was also used to investigate the oxidative desorption process of **Au–C**_**18**_**monoalkyne** and **Au–C**_**18**_**trialkyne**, as shown in Figure 7. These two modified Au electrodes exhibited a similar electrochemical oxidative behavior as **Au–C**_**18**_**alkyne**: significant oxidation peaks only appeared at a higher voltage than bare gold oxidation, more specifically, ∼1.40 V for **Au–C**_**18**_**monoalkyne** and ∼1.48 V for **Au–C**_**18**_**trialkyne**, while the reductive peaks of oxidized gold appeared during the reverse sweep indicating that the oxidation of the monolayers themselves is accompanied by Au oxidation. Our results demonstrate that these functionalized alkyne adsorbates possess an electrochemical potential window similar to alkyl alkyne (**C**_**18**_**alkyne**), which enables their application in a wide range of biosensors, and thereby their integration with recognition units (like enzymes) and attaching antifouling coatings through surface-initiated polymerization methods.

## 4 Conclusions

Detailed comparative studies reveal that monovalent alkyne-based monolayers on Au can rival their thiol-based analogs in terms of hydrolytic stability upon prolonged immersion (7 days) in four different aqueous media. Moreover, they also possess similar thermal stability when heated for 1 h at an elevated temperature (80 °C). Additionally, the multivalent effect of the tridentate structure was found to starkly improve both long-term hydrolytic stability and thermal stability of alkyne-based monolayers. Moreover, alkyne-based and thiolate monolayers exhibit higher oxidation potentials than bare Au electrodes, enabling their further application in practical biosensing when combined with recognition units, such as enzymes or antibodies, allowing for real-world sensing in complex media such as bioreactors or bodily fluids. Overall, our findings underscore the advantages of terminal alkyne SAMs in sensor technology. Particularly, the integrated capability of their long-term hydrolytic stability, thermal stability, the reported efficient electron transfer, integration with various recognition units and inertness toward (bio)thiols substitution all position alkyne-based monolayers as valuable candidates for advanced biosensor development.

## Supporting Information Available

Detailed synthetic protocols and characterization data (^1^H and ^13^C NMR spectrum and MS) for adsorbates; diagram for surface cleaning method comparison; diagram for thermal stability of **Au–C**_**18**_**SH** and **Au–C**_**18**_**alkyne** at various temperatures; and XPS C 1s narrow spectra for different monolayers (PDF)
