# Supplementary material for: Hydrolytic, Thermal, and Electrochemical Stability of Thiol- and Terminal Alkyne-Based Monolayers on Gold: A Comparative Study
Source: Langmuir. 2025 Mar 1;41(9):6197–207. doi: 10.1021/acs.langmuir.4c05211 (PMC11912541; doi:10.1021/acs.langmuir.4c05211)
Supplement: Supplementary file 1 — la4c05211_si_001.pdf [file la4c05211_si_001.pdf]

Supporting Information for:

Hydrolytic, thermal and electrochemical stability of thiol- and terminal alkyne-based monolayers on gold: a comparative study

Zhen Yang,[a,b] Sidharam P. Pujari,[b] Rachel Armstrong,[a] Klaus Mathwig,[a] Floris P. J. T. Rutjes,[e] Maarten M. J. Smulders,[b*] and Han Zuilhof[b,c,d*]

[a] *imec within OnePlanet Research Center, Bronland 10, 6708 WH Wageningen, The Netherlands*

[b] *Laboratory of Organic Chemistry, Wageningen University & Research, Stippeneng 4, 6708WE Wageningen, The Netherlands*

[c] *School of Pharmaceutical Sciences and Technology, Tianjin University, 92 Weijin Road, Tianjin 300072, P. R. China*

[d] *College of Biological and Chemical Engineering, Jiaxing University, Jiaxing 314001, P. R. China*

[e] *Institute for Molecules and Materials, Radboud University, Heyendaalseweg 135, 6525 AJ Nijmegen, The Netherlands*

* maarten.smulders@wur.nl, han.zuilhof@wur.nl

Table of content

S1. Synthesis and characterization S3

S2. NMR spectra S5

S3. Surface cleaning methods S9

S4. Thermal stability of **Au-C_{18}SH** and **Au-C_{18}alkyne** S9

S5. XPS spectra S10

S6. References S14

S1. Synthesis and characterization

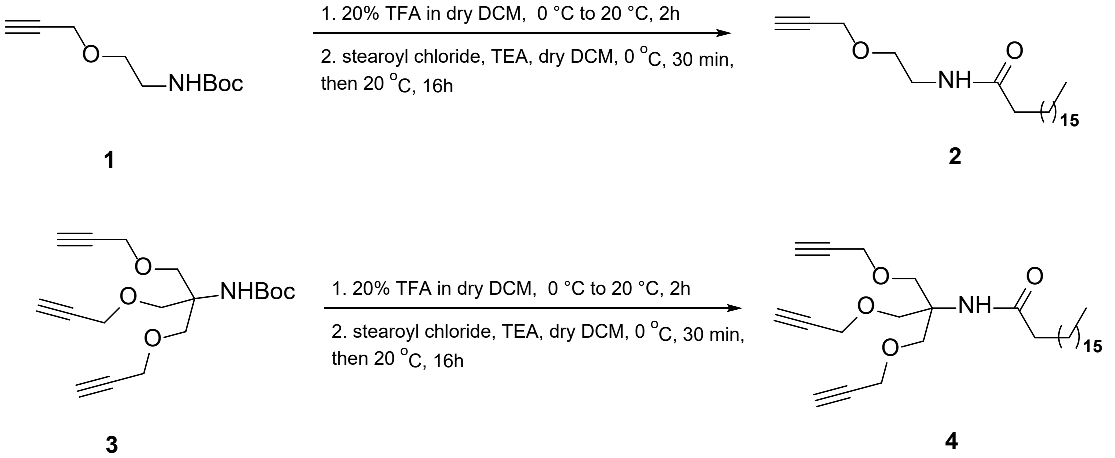

Scheme S1. Synthesis of **C_{18}monoalkyne** (**2**) or **C_{18}trialkyne** (**4**).

*The synthesis of tert-butyl (2-(prop-2-yn-1-yloxy)ethyl)carbamate (Compound **1**):* Compound **1** was synthesized and characterized according to a reported literature procedure.[S1]

*The synthesis of Compound **2** (N-(2-(prop-2-yn-1-yloxy)ethyl)stearamide, **C_{18}monoalkyne**)*: Compound **1** (300 mg, 1.50 mmol) was dissolved in 8 mL of dry dichloromethane (DCM) followed by the slow addition of 2 mL of trifluoroacetic acid (TFA) to the solution at 0 °C. The mixture was stirred at 20 °C for 2 hours. After the reaction, the solvent and excess TFA were removed by rotary evaporation. The residue was vacuumed for 20 minutes. Next, the mixture was redissolved in 10 ml of dry DCM and triethylamine (TEA) (405 mg, 4.0 mmol) was added to the solution at 0 °C under an argon atmosphere. Subsequently, stearoyl chloride (545 mg, 1.80 mmol) was added in portions to the mixture, maintaining an argon atmosphere. The mixture was stirred at 0 °C for 30 mins and 20 °C for 16h, followed by quenching the mixture with 20 mL of 1 M NaOH and extracted with 3×10 mL of DCM. The organic phase was collected and dried with Na_2SO_4. After the filtration, the organic solvent was removed by rotatory evaporation, followed by column chromatography (PE/EA: 10/1 to 2/1), then the obtained crude product was recrystallized in HPLC-grade *n*-hexane to obtain the pure compound **2** (431 mg, 76%) as a white powder.

^{1}H NMR (400 MHz, $CDCl_3$) δ 5.79 (s, 1H), 4.16 (d, *J* = 2.4 Hz, 2H), 3.60 (dd, *J* = 5.6, 4.5 Hz, 2H), 3.48 (q, *J* = 5.3 Hz, 2H), 2.45 (t, *J* = 2.4 Hz, 1H), 2.17 (dd, *J* = 8.4, 6.9 Hz, 2H), 1.62 (p, *J* = 7.3 Hz, 2H), 1.25 (s, 28H), 0.88 (t, *J* = 6.8 Hz, 3H).

^{13}C NMR (101 MHz, $CDCl_3$) δ 173.36, 79.52, 74.88, 69.05, 58.46, 39.17, 36.96, 32.07, 29.84, 29.82, 29.80, 29.77, 29.65, 29.51, 29.44, 25.88, 22.83, 14.26.

HRMS (ESI) m/z $[M+Na]^+$ Calcd. for $C_{23}H_{43}NO_2Na$: 388.319, found 388.319.

The synthesis of Compound 3: Compound **3** was synthesized according to a reported literature procedure.[S2]

The synthesis of *Compound 4 (N-(1,3-bis(prop-2-yn-1-yloxy)-2-((prop-2-yn-1-yloxy)methyl)propan-2-yl)stearamide ,* ***C_{18}trialkyne***): Compound **3** (300 mg, 0.89 mmol) was dissolved in 8 mL of dry dichloromethane (DCM) followed by the slow addition of 2 mL of trifluoroacetic acid (TFA) to the solution at 0 °C. The mixture was stirred at 20 °C for 2 hours. After the reaction, the solvent and excess TFA were removed by rotary evaporation. The residue was vacuumed for 20 minutes. Next, the mixture was redissolved in 10 ml of dry DCM and TEA (240 mg, 2.37 mmol) was added to the solution at 0 °C under an argon atmosphere. Subsequently, stearoyl chloride (324 mg, 1.07 mmol) was added in portions to the mixture, maintaining an argon atmosphere. The mixture was stirred at 0 °C for 30 mins and 20 °C for 16h, followed by quenching the mixture with 20 mL of 1M NaOH and extracted with 3×10 mL of DCM. The organic phase was collected and dried with Na_2SO_4, After the filtration, the organic solvent was removed by rotatory evaporation, followed by column chromatography (PE/EA: 10/1 to 5/1), then the obtained crude product was recrystallized in HPLC-grade *n*-hexane to get the pure compound **4** (237 mg, 53%) as a white powder.

^{1}H NMR (400 MHz, $CDCl_3$) δ 5.67 (s, 1H), 4.15 (d, J = 2.4 Hz, 6H), 3.85 (s, 6H), 2.43 (t, J = 2.4 Hz, 3H), 2.14 (t, J = 7.6 Hz, 2H), 1.59 (t, J = 7.2 Hz, 2H), 1.25 (s, 28H), 0.88 (t, J = 6.7 Hz, 3H).

^{13}C NMR (101 MHz, $CDCl_3$) 173.56, 79.74, 74.72, 68.77, 59.25, 58.81, 37.56, 32.07, 29.85, 29.82, 29.80, 29.79, 29.67, 29.54, 29.51, 29.30, 25.77, 22.84, 14.27.

HRMS (ESI) m/z $[M+Na]^+$ Calcd. for $C_{31}H_{51}NO_4Na$ for 524.372, found 524.372.

S2. NMR spectra

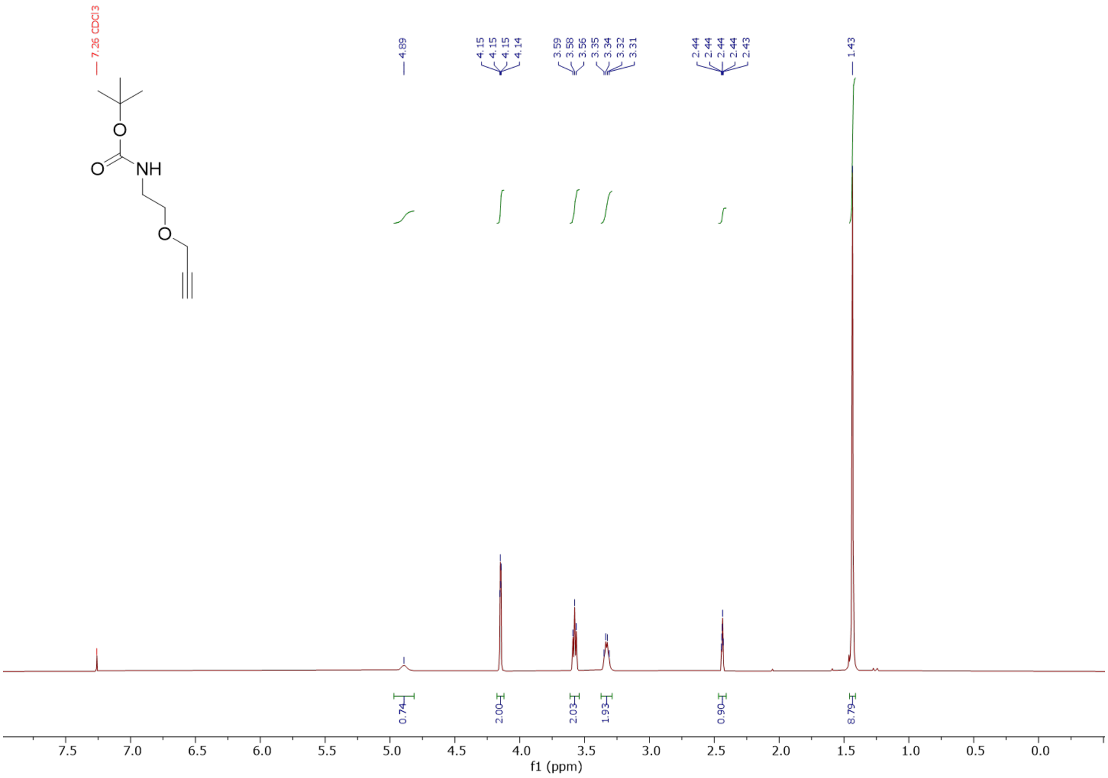

Figure S1. ^{1}H NMR spectrum of compound **1** (400 MHz, $CDCl_3$, 298K).

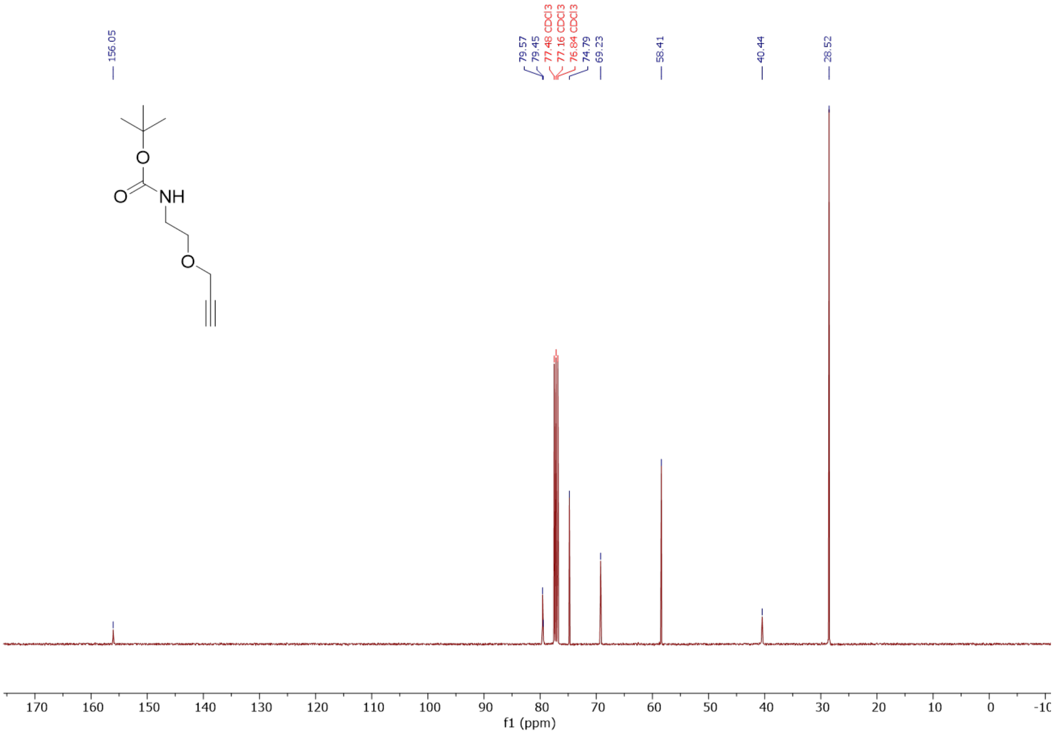

Figure S2. ^{13}C NMR spectrum of compound **1** (101 MHz, $CDCl_3$, 298K).

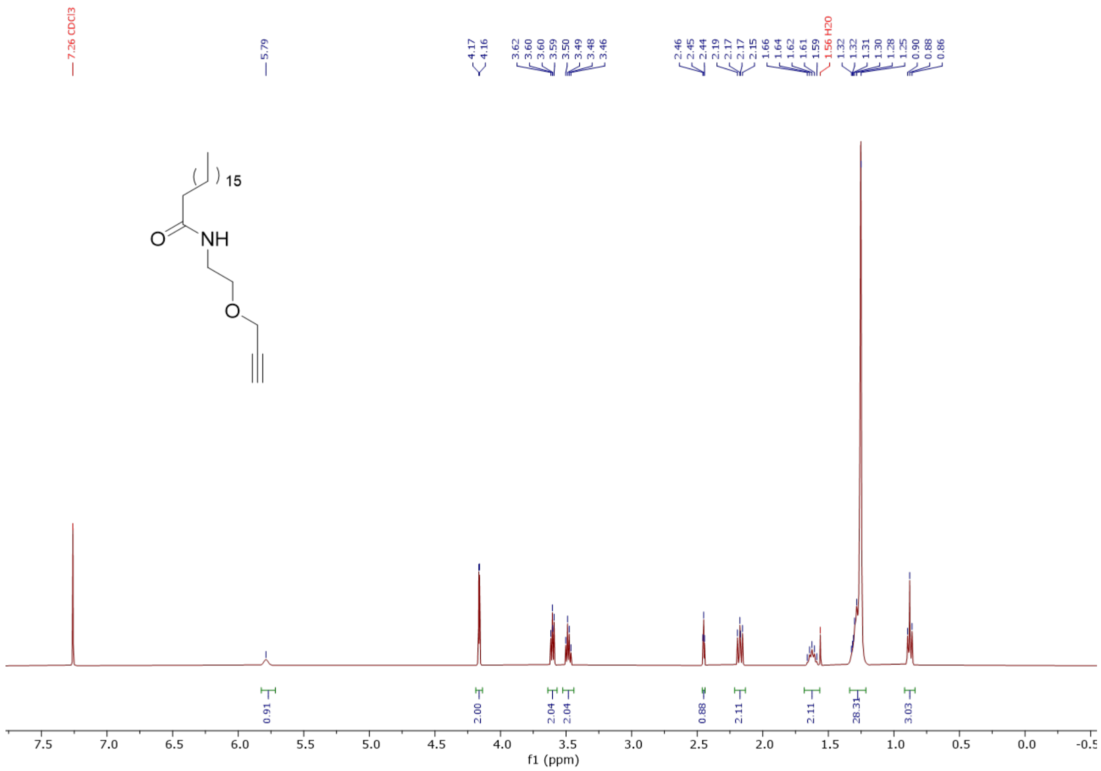

Figure S3. ^{1}H NMR spectrum of compound **2** (400 MHz, $CDCl_3$, 298K).

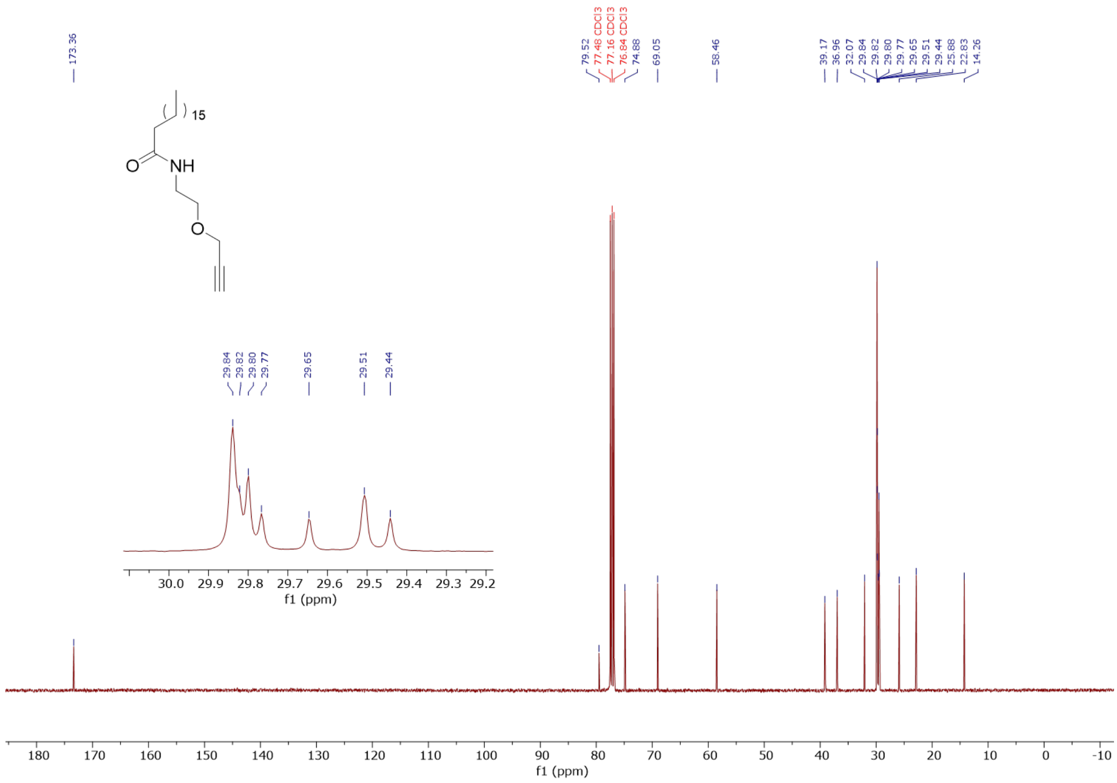

Figure S4. ^{13}C NMR spectrum of compound **2** (101 MHz, $CDCl_3$, 298K).

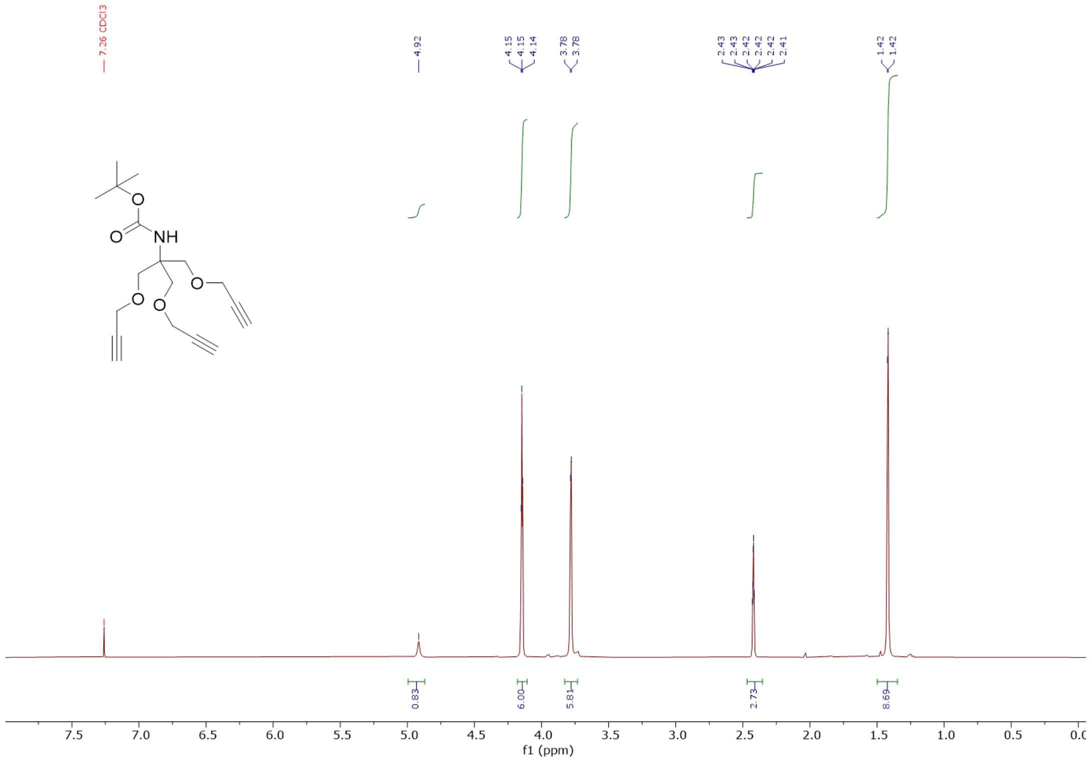

Figure S5. ^{1}H NMR spectrum of compound **3** (400 MHz, $CDCl_3$, 298K).

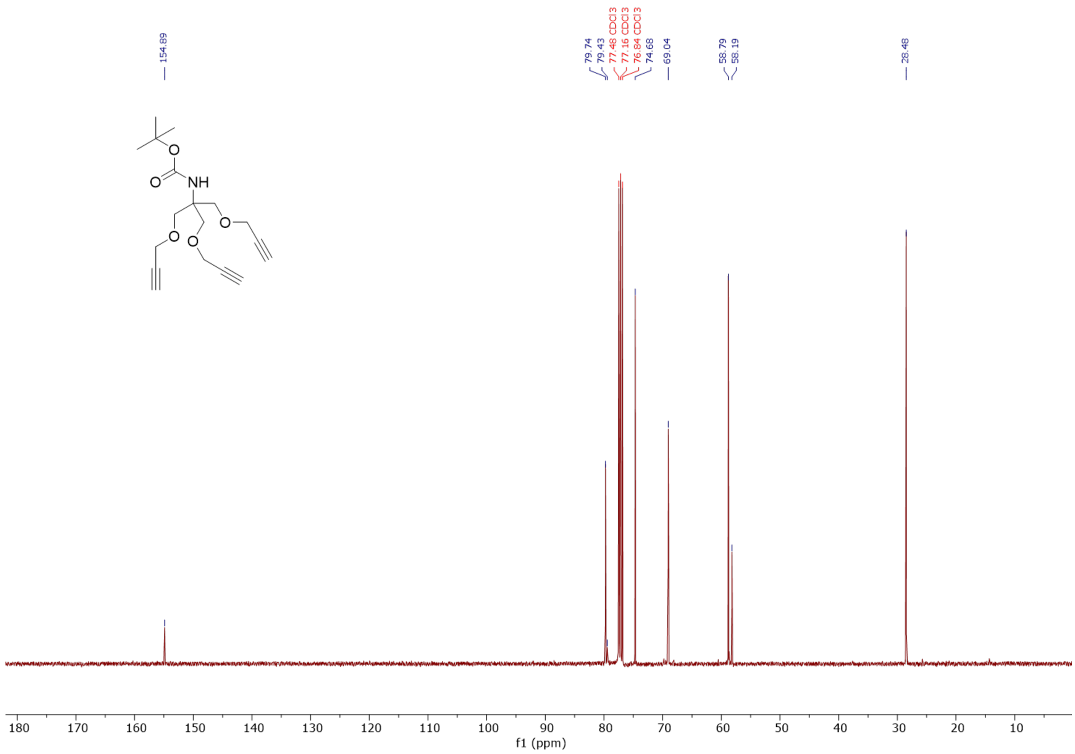

Figure S6. ^{1}H NMR spectrum of compound **3** (101 MHz, $CDCl_3$, 298K).

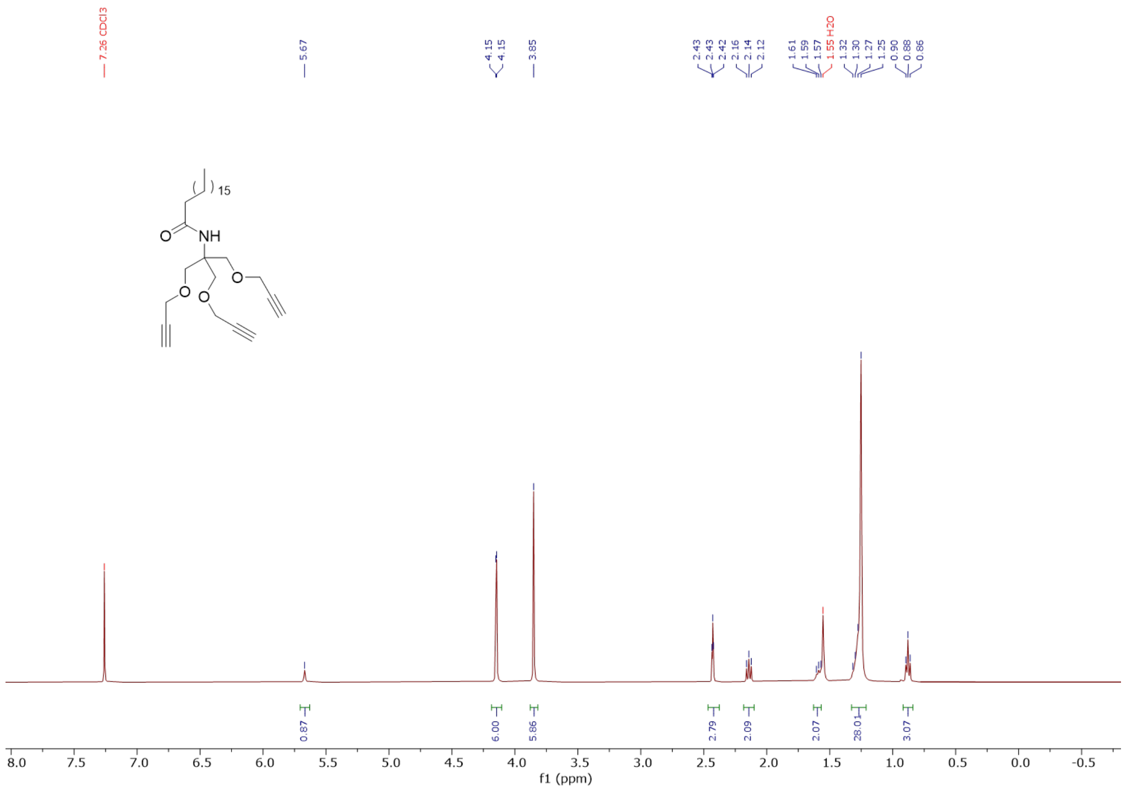

Figure S7. ^{1}H NMR spectrum of compound **4** (400 MHz, $CDCl_3$, 298K).

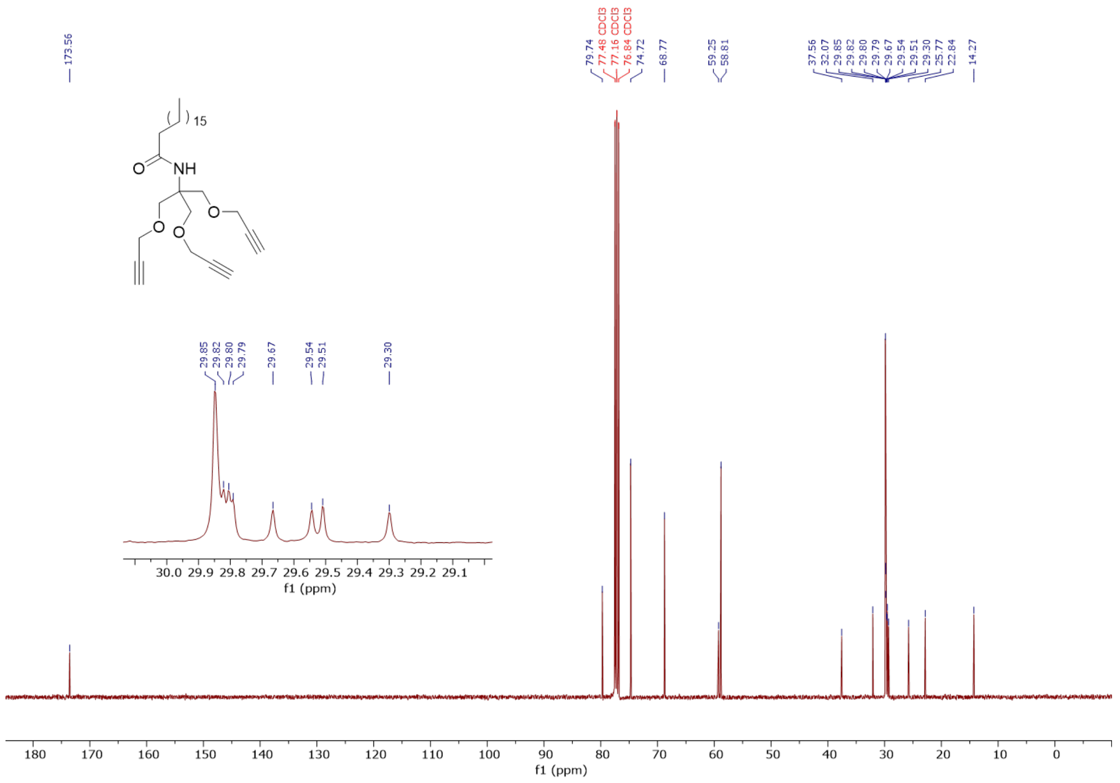

Figure S8. ^{13}C NMR spectrum of compound **4** (101 MHz, $CDCl_3$, 298K).

S3. Surface cleaning methods

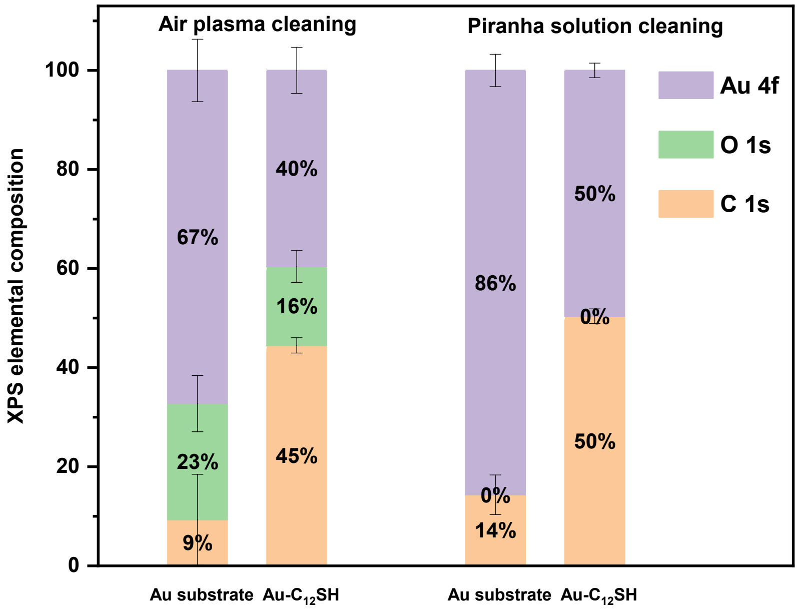

Figure S9. XPS elemental percentage comparison of gold substrates cleaned with piranha solution or air plasma (cleaning time was 10 min in both cases) and the corresponding **Au-C$_{12}$SH**.

S4. Thermal stability of Au-C$_{18}$SH and Au-C$_{18}$alkyne

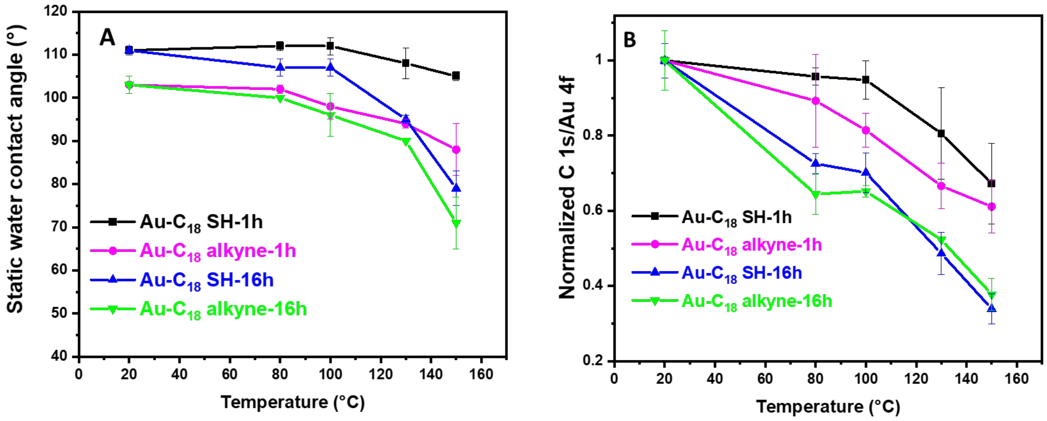

Figure S10. (A) SWCA and (B) normalized XPS C 1s/Au 4f signal ratio for **Au-C$_{18}$SH** and **Au-C$_{18}$alkyne** at indicated heating temperature for 1 h and 16 h.

S5. XPS spectra

Below XPS C 1s narrow scan spectra for the various tested monolayers are depicted.

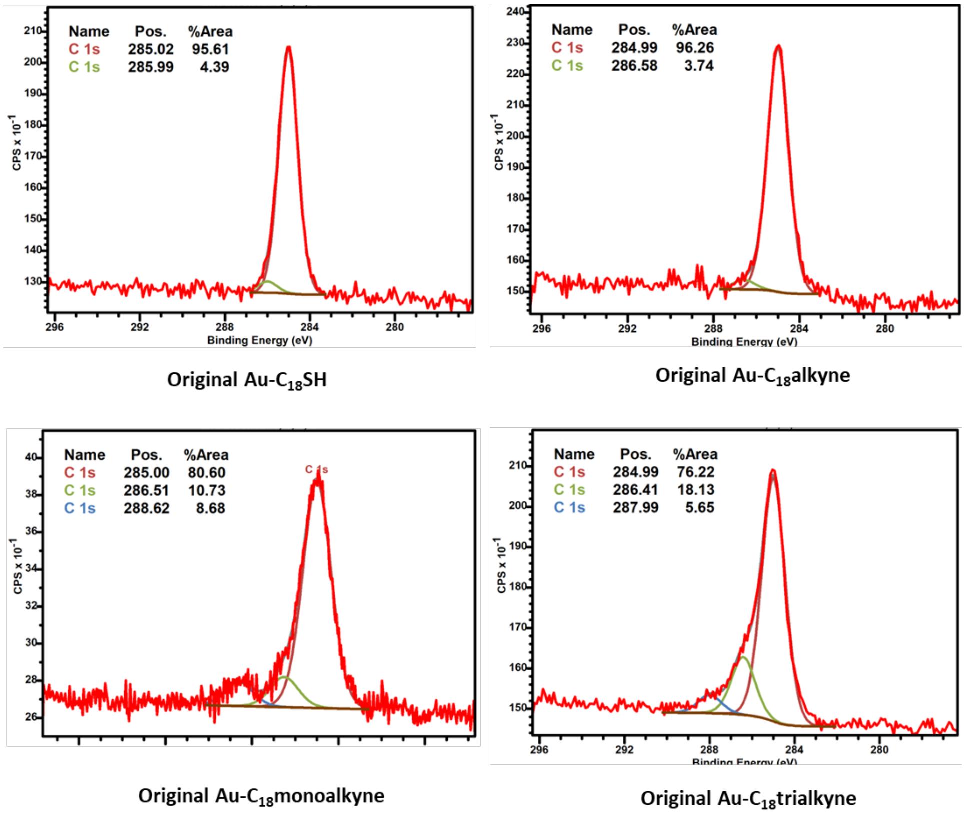

Figure S11. XPS C 1s narrow scan spectra for the original **Au-C$_{18}$SH**, **Au-C$_{18}$alkyne Au-C$_{18}$monoalkyne,** and **Au-C$_{18}$trialkyne** monolayers.

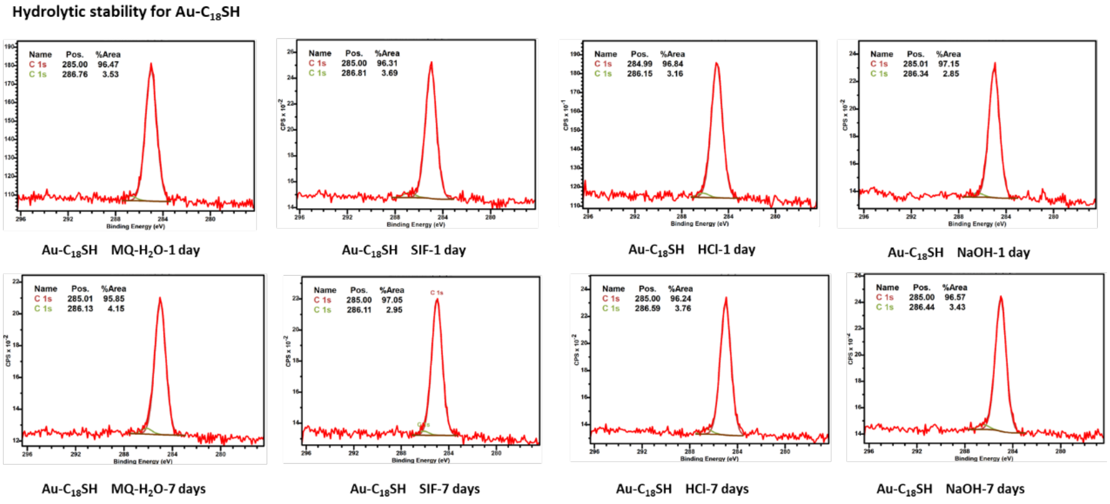

Figure S12. XPS C 1s narrow scan spectra for the hydrolytic stability test of the **Au-C$_{18}$SH** monolayer.

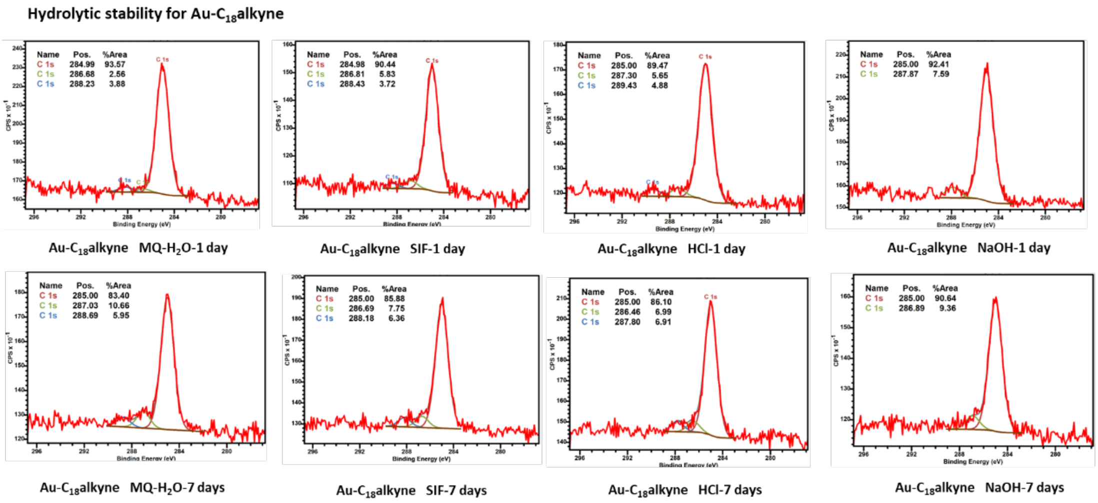

Figure S13. XPS C 1s narrow scan spectra for the hydrolytic stability test of the **Au-C18alkyne** monolayer.

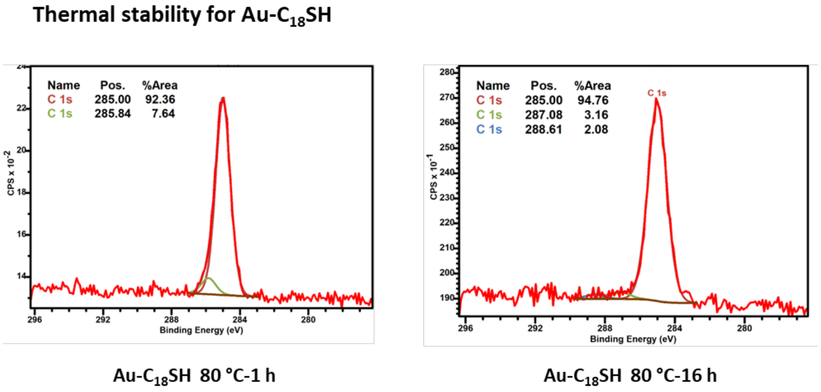

Figure S14. XPS C 1s narrow scan spectra for the thermal stability test of the **Au-C18SH** monolayer.

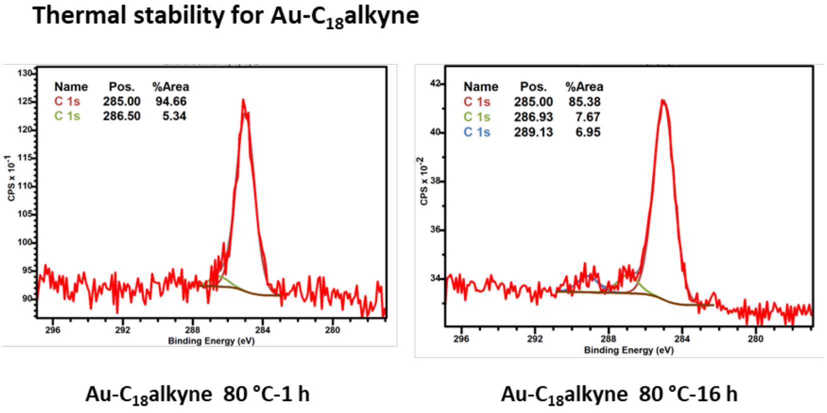

Figure S15. XPS C 1s narrow scan spectra for the thermal stability test of the **Au-C18alkyne** monolayer.

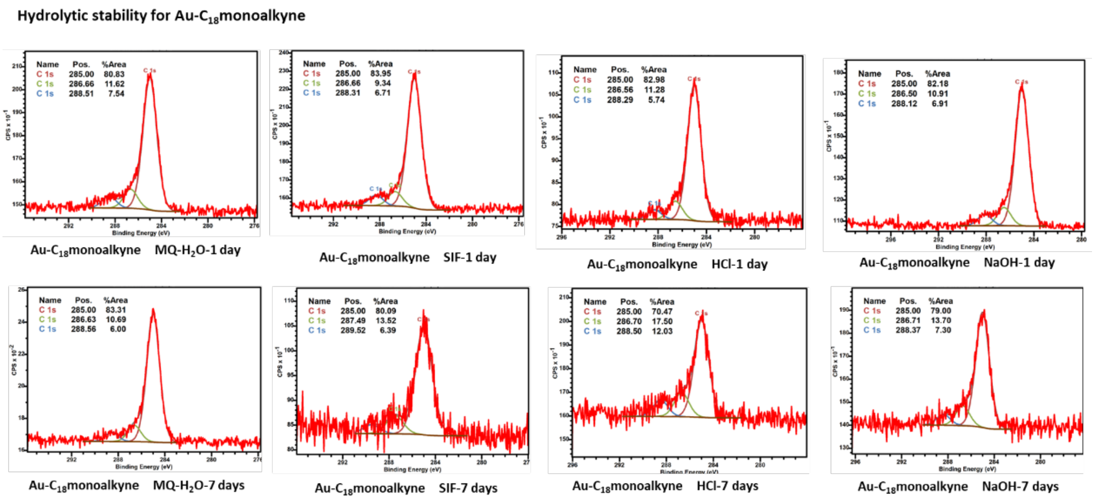

Figure S16. XPS C 1s narrow scan for the hydrolytic stability test of the **Au-C₁₈monoalkyne** monolayer.

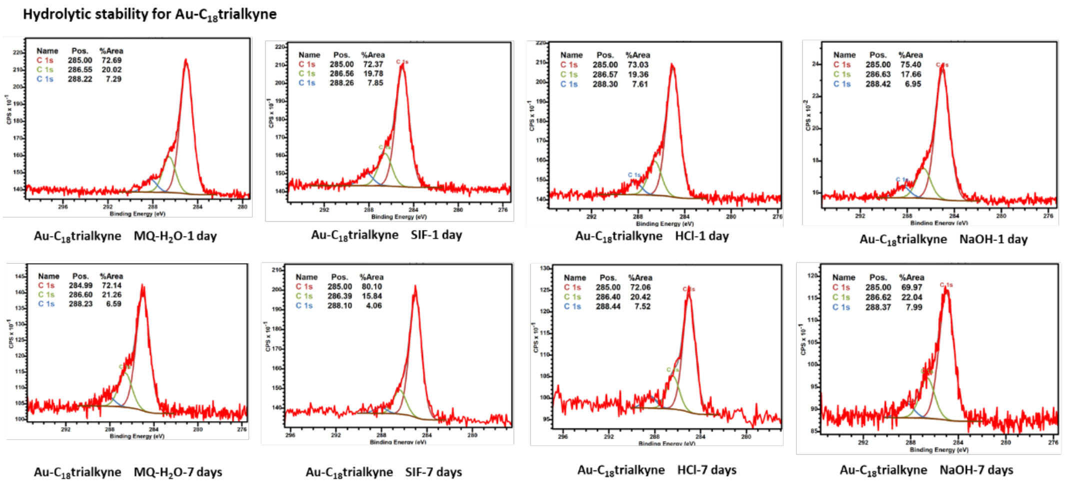

Figure S17. XPS C 1s narrow scan spectra for the hydrolytic stability test of the **Au-C₁₈trialkyne** monolayer.

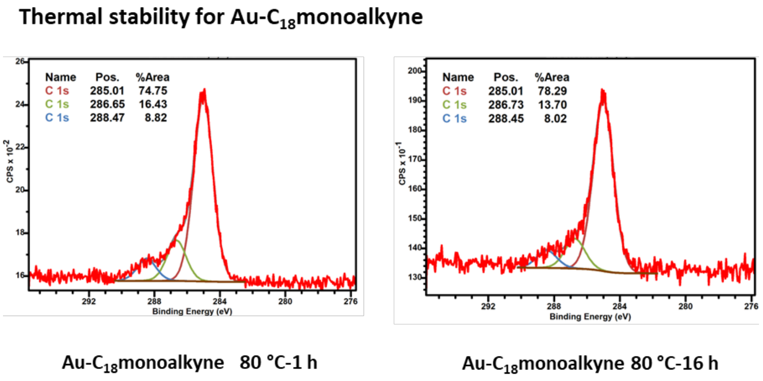

Figure S18. XPS C 1s narrow scan spectra for the thermal stability test of the **Au-C₁₈monoalkyne**

Thermal stability for Au-C_{18}trialkyne

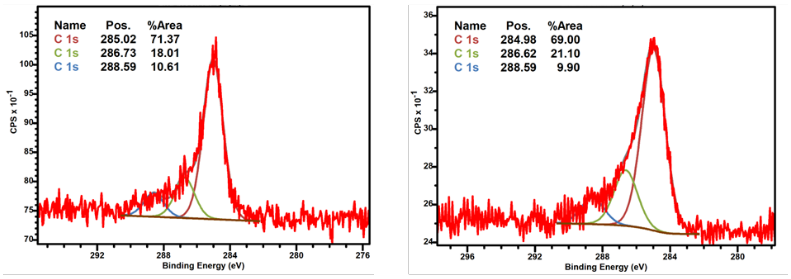

Au-C_{18}trialkyne 80 °C-1 h   Au-C_{18}trialkyne 80 °C-16 h

Figure S19. XPS C 1s narrow scan spectra for the thermal stability test of the **Au-C_{18}trialkyne** monolayer.

S6. References

(S1) Chang, T. C.; Lai, C. H.; Chien, C. W.; Liang, C. F.; Adak, A. K.; Chuang, Y. J.; Chen, Y. J.; Lin, C. C. Synthesis and Evaluation of a Photoactive Probe with a Multivalent Carbohydrate for Capturing Carbohydrate-Lectin Interactions. *Bioconjugate Chem.* 2013, *24* (11), 1895-1906. DOI: 10.1021/bc400306g.

(S2) Chabre, Y. M.; Contino-Pépin, C.; Placide, V.; Shiao, T. C.; Roy, R. Expeditive synthesis of glycodendrimer scaffolds based on versatile TRIS and mannoside derivatives. *J. Org. Chem.* 2008, *73* (14), 5602-5605. DOI: 10.1021/jo8008935.
